# Elucidating the multiple roles of hydration for accurate protein-ligand binding prediction via deep learning

**DOI:** 10.1038/s42004-020-0261-x

**Published:** 2020-02-11

**Authors:** Amr H. Mahmoud, Matthew R. Masters, Ying Yang, Markus A. Lill

**Affiliations:** 1 Department of Medicinal Chemistry and Molecular Pharmacology, College of Pharmacy, Purdue University, 575 Stadium Mall Drive, West Lafayette, IN 47906 USA; 2 Department of Pharmaceutical Sciences, University of Basel, Klingelbergstrasse 50, 4056 Basel, Switzerland

**Keywords:** Structure-based drug design, Molecular modelling, Computational chemistry

## Abstract

Accurate and efficient prediction of protein-ligand interactions has been a long-lasting dream of practitioners in drug discovery. The insufficient treatment of hydration is widely recognized to be a major limitation for accurate protein-ligand scoring. Using an integration of molecular dynamics simulations on thousands of protein structures with novel big-data analytics based on convolutional neural networks and deep Taylor decomposition, we consistently identify here three different patterns of hydration to be essential for protein-ligand interactions. In addition to desolvation and water-mediated interactions, the formation of enthalpically favorable networks of first-shell water molecules around solvent-exposed ligand moieties is identified to be essential for protein-ligand binding. Despite being currently neglected in drug discovery, this hydration phenomenon could lead to new avenues in optimizing the free energy of ligand binding. Application of deep neural networks incorporating hydration to docking provides 89% accuracy in binding pose ranking, an essential step for rational structure-based drug design.

## Abstract (web summary)

Accurate prediction of protein-ligand binding is limited by our ability to model solvation. Here, in addition to protein and ligand information, density maps for hydration site occupancy are used to train a convolutional neural network in order to predict binding poses more accurately compared to common scoring functions.

## Introduction

Recently, convolutional neural networks (CNNs) have been widely applied to different fields in computational chemistry such as QSAR, virtual screening, de novo molecular design, quantum chemistry, prediction of molecular properties, and materials design^1–4^. Several groups utilized CNNs to generalize the modeling of protein-ligand interactions in context of scoring functions^5,6^. In these approaches, the structural information of a protein-ligand complex is provided to the CNN as three-dimensional (3D) images representing the density of different atom types. The architecture of CNNs allows them to learn from these data important local features of protein-ligand interactions, without limitation to pre-defined scoring functions that typically rely on two-body interaction models. Current CNN models are focused on modeling the direct interactions between protein and ligand in the bound state, but neglect critical contributions of protein-ligand binding, in particular the solvation and desolvation of protein and ligand upon binding.

**Fig. 1:**
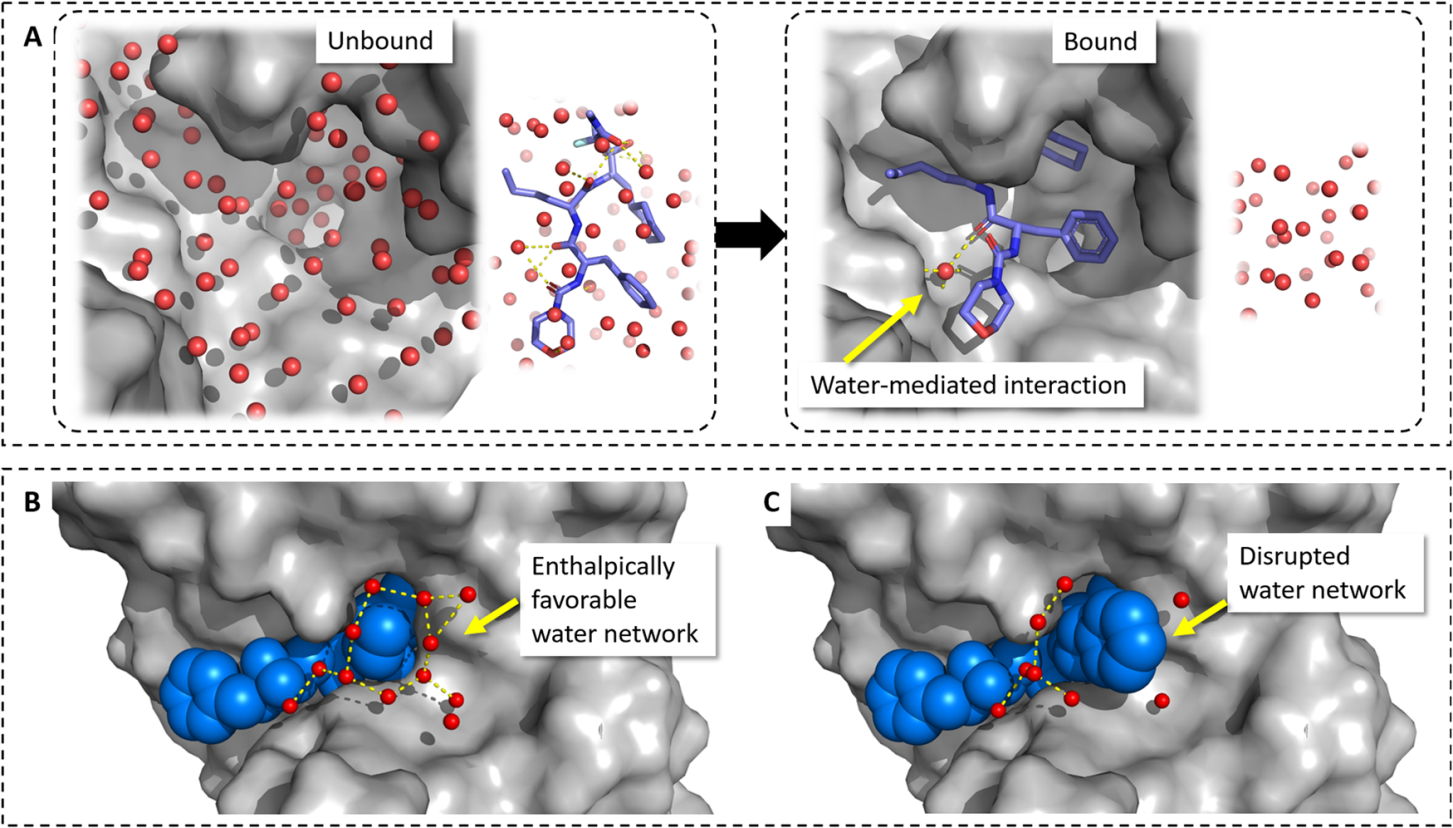
Types of hydration essential for protein-ligand binding. **a** Protein and ligand desolvation, in particular of apolar moieties is usually an essential driving force for ligand binding. Polar ligand and protein groups can interact via mediating water molecules. **b** The formation of enthalpically stable first-shell water layers is still a widely unrecognized hydration phenomena important for protein-ligand binding. Disruption of water network (**c**) can lead to significant reduction in binding enthalpy^25^.

Water is a crucial player in protein-ligand binding processes (Fig. 1)^7,8^. In structure-based drug design, ligands are optimized to replace energetically unfavorable water molecules, particularly ordered water molecules in hydrophobic moieties. This desolvation of free energy is often an essential driving force for strong protein-ligand association (Fig. 1a). In contrast, enthalpically favorable water molecules are often utilized as part of the ligand design process as mediator of protein-ligand interactions (Fig. 1a).

Traditionally, desolvation effects in protein-ligand scoring were modeled by an empirical term characterizing hydrophobic contacts between the protein and the ligand, for example, using counts of close hydrophobic protein-ligand atom pairs or using a term that is proportional to the solvent-accessible surface^9–12^. These approaches are implicitly modeling desolvation effects, but ignore thermodynamic properties of individual binding site water molecules, which can significantly differ based on the detailed protein environment (Supplementary Note 1)^8,13,14^.

To explicitly and more precisely model solvation effects of individual water molecules, a spectrum of various computational approaches have been developed to identify the likely position of water molecules in binding sites and to evaluate their energetic stability^15^. Accurate estimation of position and desolvation energies of individual water molecules can be obtained by Monte-Carlo (MC) or molecular-dynamics (MD)-based simulation methods, such as WaterMAP^8,16^ or WATsite^13,14^. Considering the excess enthalpy and entropy of the water molecules in the binding site that are replaced by a ligand, the contribution of desolvation to the binding free energy can in principle be computed^8,17,18^. For a direct inclusion of explicit desolvation into standard scoring procedures, grid-based adaptations of the inhomogeneous solvation theory (IST)^19,20^, such as GIST^21^, were utilized. Even with such accurate desolvation estimation, the docking performance did not significantly nor consistently improve for most protein targets^22^. Reasons for these results include the lack of consistent scoring function design and optimization, including the explicit desolvation term (desolvation term has been typically used as a subsequent add-on to existing scoring functions), inaccuracies in the other interaction terms, and the neglect of water-mediated interactions.

**Fig. 2:**
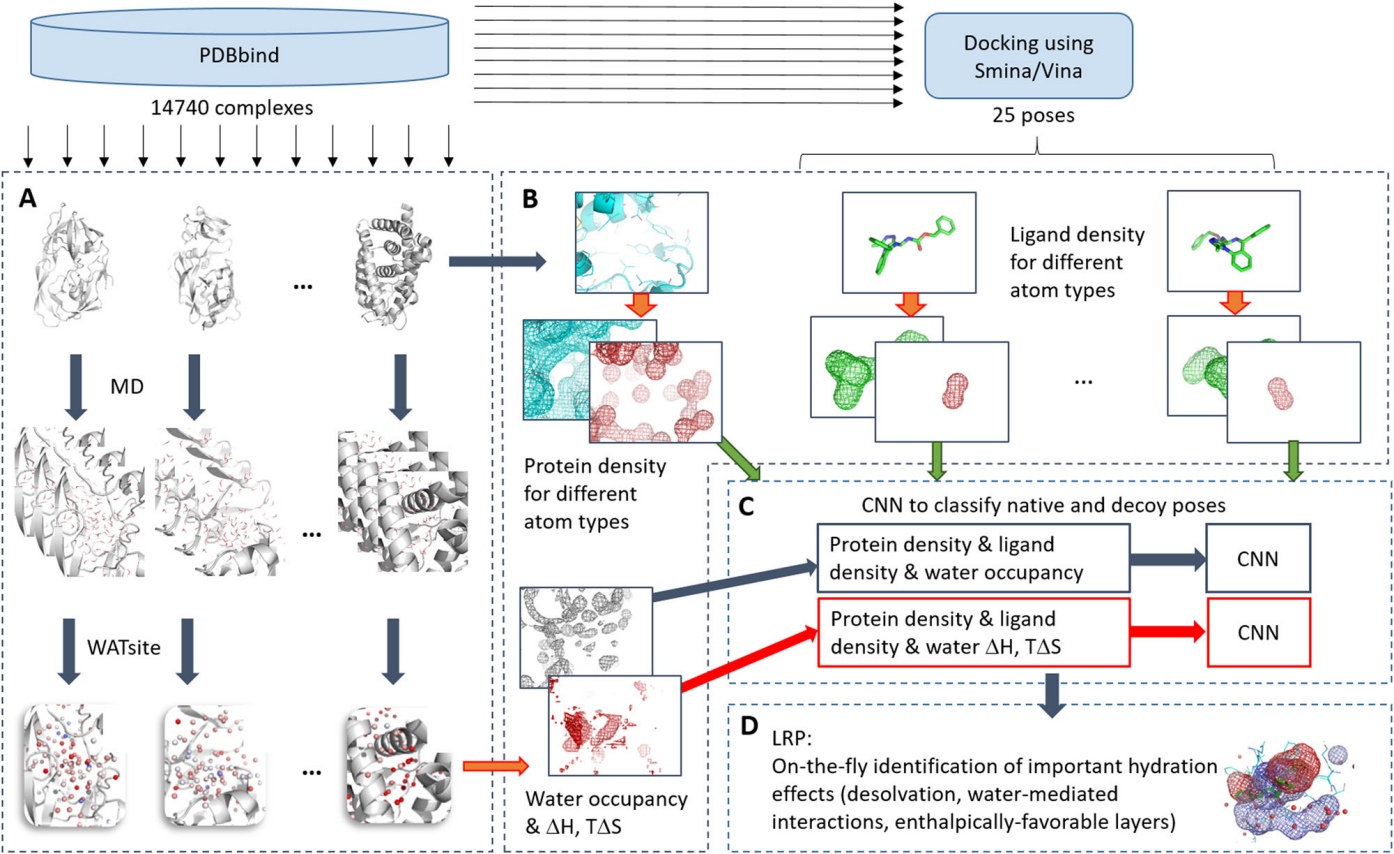
Overall procedure of DeepWATsite. **a** Hydration information for 14,740 protein structures from PDBbind was generated using a combination of 3D-RISM, GAsol, MD, and WATsite^26^. **b** Hydration occupancy, enthalpy, and entropy information was distributed on a 3D grid encompassing the binding site. Protein and ligand density for all poses was distributed on a 3D grid with same dimensions using Gaussian distribution functions. **c** All 3D grids were used as different channels of the input layer of a CNN model. The model was trained to separate active from decoy poses. **d** In addition to significant improvement in pose ranking, the underlying CNN model identified on-the-fly important hydration contributions to ligand binding.

**Fig. 3:**
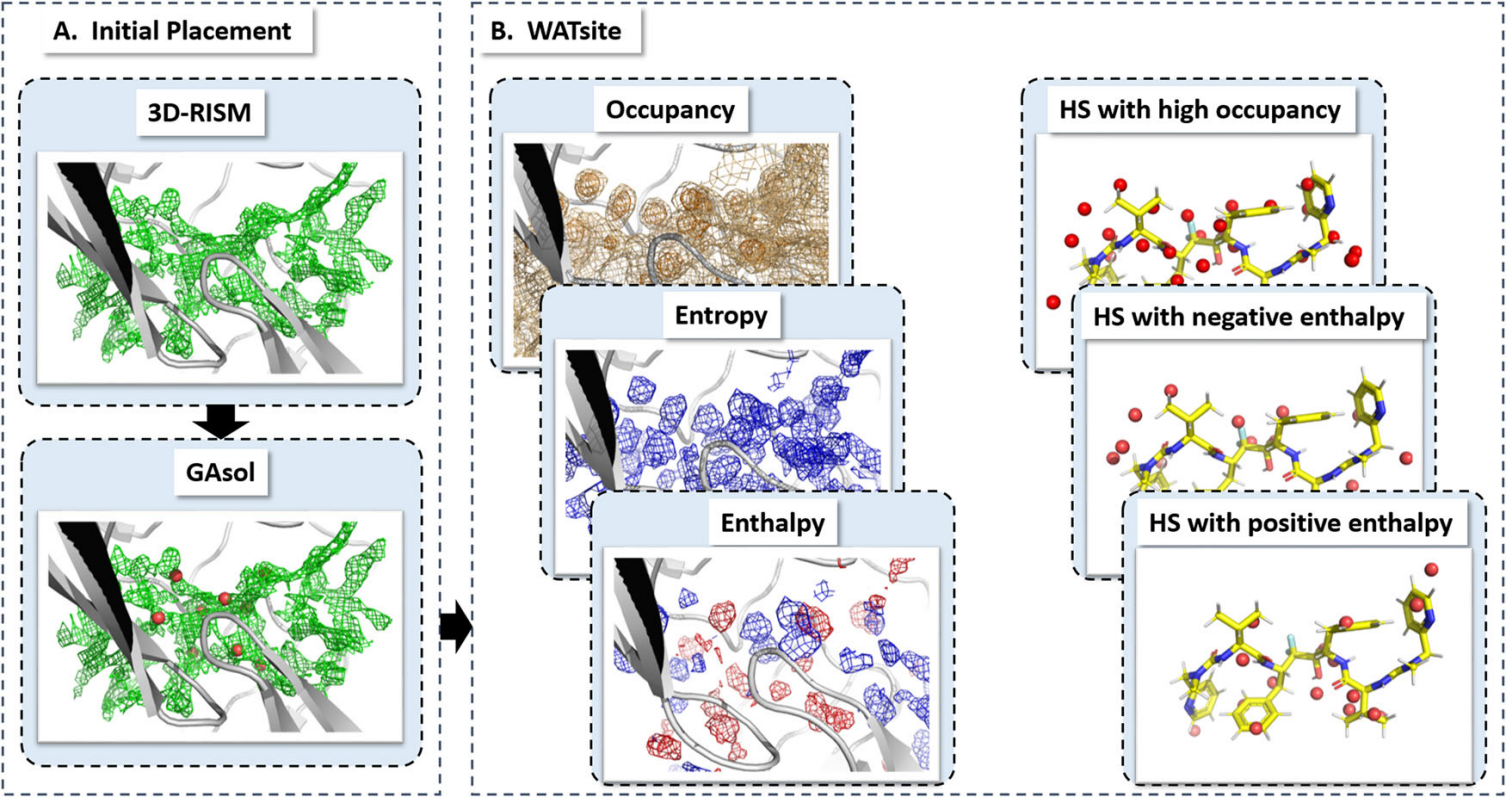
Overall procedure of WATsite. **a** Initial placement of water molecules using 3D-RISM and GAsol. **b** Subsequent MD simulation with explicit water molecules and WATsite analysis generating water-occupancy, enthalpy, and entropy grids. Hydration data (here in form of sites) can be used to understand the effect of (de)solvation on protein-ligand thermodynamics.

Here we present to the best of our knowledge the first attempt to include explicit hydration information into CNN-based scoring, titled DeepWATsite (Fig. 2), which exhibits significant improvement in binding pose ranking. The CNN model utilizes protein and ligand density as well as hydration information as input (Fig. 2a, b). Protein and ligand atomic densities for each pose as well as water-occupancy or thermodynamic data are distributed on a 3D grid encompassing the binding site (Fig. 2b). Those layers of 3D images are presented as input to the CNN that learns to separate native from incorrect poses (Fig. 2c). Dependent on the utilized hydration data (occupancy or thermodynamic values), different CNN models are generated. To train and test CNN models, here for the development of a scoring function, a large data set of protein-ligand complex structures is required. With respect to the calculation of hydration data for a large set of protein systems, the underlying molecular-dynamics (MD) simulations for WATsite on the ligand-free form of the protein system (Figs. 2a and 3) are rather time consuming. To apply the method to a large number of protein structures, we have developed a new accelerated WATsite implementation based on graphics processing unit (GPU) acceleration, asynchronous data output, and protein truncation (for details see Methods and Supplementary Methods sections).

A common criticism of deep neural networks is the “black-box” character of the generated models. To allow for a direct interpretation of the CNN models, we implemented a recently developed form of layer-wise relevance propagation (LRP) methodology based on deep Taylor decomposition^23^ (Fig. 2d). The LRP analysis reveals that DeepWATsite is able to identify and model water-mediated interactions and desolvation contributions to protein-ligand binding. Furthermore, an additional hydration phenomenon critical for protein-ligand binding is revealed, enthalpically favorable first-shell hydration layers around solvent-exposed ligand moieties (Fig. 1b)^24,25^.

## Results and Discussion

### Initial CNN models

Hydration site data was generated for several thousand protein systems using the new GPU-accelerated version of WATsite (see Methods and Supplementary Methods sections). Our recent protocol combining 3D-RISM, GAsol, and WATsite (Fig. 3) was utilized to achieve convergence for hydration site occupancy and thermodynamics predictions even for occluded binding sites^26^. In short, water molecules were initially placed around a protein surface using 3D-RISM site-distribution function^27–29^ and GAsol^30^ to optimize the initial water network. Using this initial water placement, WATsite then performs explicit water MD simulations of each protein and computes explicit water-occupancy and free-energy profiles of each hydration site (=high water-occupancy spot) in the binding site.

This hydration data is distributed on a 3D grid encompassing the binding site and can be viewed as 3D images. In addition to protein and ligand atom-type densities (also distributed on the same 3D grid), these images are considered as input to the subsequent CNN models (Fig. 2b, c). The CNN models learn features from the input layers using three convolutional layers, including max pooling, and finally use those features to predict the correctness of a binding pose using a fully connected and a softmax layer (for details see Methods section).

First, the CNN models were trained on a rather small set of 377 protein systems and validated on a much larger test set of 2046 systems demonstrating the universality of the generated models. These models are based on 25 poses per protein-ligand system, the typical output from docking, here using Smina. Systems for training and test set were randonly selected from PDBbind. The test set displays high diversity in ligand chemistry and protein structure. It includes structures from 369 protein families based on PFAM classification (see Supplementary Table 1).

Three different CNN models were generated for comparison purposes, one with only occupancy information about hydration (named DeepWATsite(occ, small)), one with positive and negative enthalpy as well as entropy information about hydration (named DeepWATsite(H−, H+, TS, small)), and one without any hydration information (named CNN(P + L)) (see Methods).

**Fig. 4:**
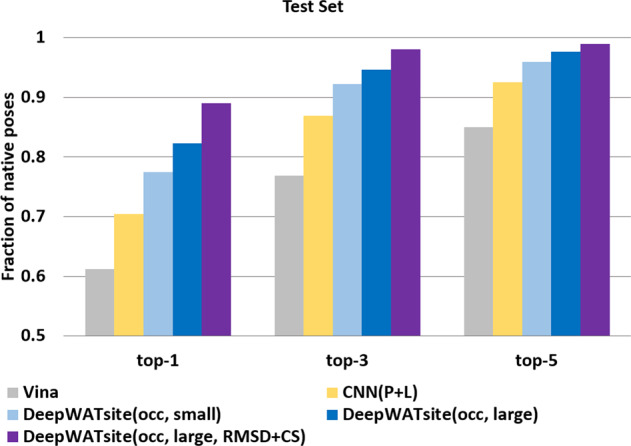
Pose ranking accuracy. Percentage of protein systems with native pose (RMSD  <2 Å) in the test set within the top 1-, top 3-, and top 5-ranked poses using different scoring functions: Vina (gray), CNN with protein and ligand information (yellow), DeepWATsite with occupancy and small training set (light blue), DeepWATsite with occupancy and large training set (blue), and DeepWATsite with occupancy, large training set, and additional corrections, such as loss function based on modified RMSD and contact score (purple).

Figure 4 compares the number of targets with successful identification of native poses (root mean square deviation [RMSD]  <2 Å) ranked among the top 1, top 3, or top 5 by using Vina scoring function, CNN(P + L) scoring using only protein and ligand information and two of the three models of DeepWATsite, including protein, ligand, and hydration information. Only the results for the 2046 test systems are shown.

Whereas Vina identifies native poses in 61%, 77%, and 85% of all systems in the top 1-, top 3-, and top 5-ranked poses, respectively, CNN(P + L) significantly outperforms Vina in identifying native poses. CNN(P + L) displays an improvement of 9%, 10%, and 7% compared to Vina scoring in the top 1-, top 3-, and top 5-ranked poses, respectively.

Including hydration information into the CNN model further improves the prediction quality significantly. The occupancy hydration model improves the prediction of Vina by 16%, 15%, and 11%, the enthalpy/entropy model by 14%, 14%, and 11% (not shown in figure) at the top 1, top 3, and top 5 levels, respectively. If the search algorithm is able to provide a native-like pose, DeepWATsite can rank this pose within the top 5-ranked poses in almost all cases. Combining occupancy, enthalpy and entropy layers in a model did not show any further prediction quality improvements. To limit the number of input layers, all subsequent models were generated with the hydration information provided only in the form of an occupancy layer.

Considering the small size of the training set, one logical approach aiming to improve model accuracy is to increase the data size for model building. Using the general set from PDBBind, a model was trained on 12,694 protein-ligand systems^31^. Applying the model to the identical test set of 2046 systems resulted in only moderate enhancement in prediction performance from 77% to 82% in the top 1 rank (92 to 95% in top 3, 96 to 98% in top 5) (Fig. 4, DeepWATsite(occ, large)). This moderate boost despite the significant training set size increase by more than 30-fold indicates that the model performance might be affected by other factors than the training-data availability.

### Critical investigation of model failures and improved model building

As previously mentioned, a near-native pose was identified among the top 5-ranked poses for almost all systems. Nevertheless, for 16% of all systems this near-native pose could not be ranked first by the deep-learning model. Furthermore, for about 150 systems, which failed in the top rank but had a near-native pose at the second rank, the CNN score was almost identical. Further analysis revealed four main reasons for this difficulty in reproducing the native pose at the top of the ranked list.

**Fig. 5:**
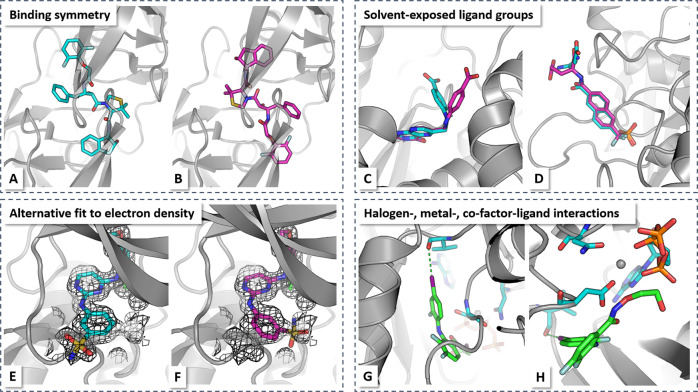
Causes for prediction failures. X-ray structure of HIV-1 protease with inhibitor KNI-10265 (PDB-code: 3kdd^58^) revealed two alternative ligand orientations (**a**, **b**) due to the local two-fold symmetry axis of the protein. Original PDBBind data only considered one of the orientations as native. **c**, **d** Solvent-exposed groups can differ between X-ray binding mode (cyan) and best docking pose (magenta) without influence on native protein-ligand contacts. Here, examples of ricin complex with pteroic acid (1br6^59^, **c**) and protein tyrosine phosphatase 1B with phosphotyrosine peptide mimetic (1bzc^60^, **d**) are shown. **e**, **f** Top-docking pose may be alternative ligand orientation fitting into experimental electron density (**e** x-ray pose: cyan; **f** docking pose: magenta). Here, example of ephB4 kinase domain inhibitor complex is shown (2vwx^61^). **g**, **h** Halogen-, metal-, co-factor-ligand interactions may be not modeled well due to **a** infrequent occurrence in training set, **b** diversity in coordination of metal-ligand interactions, or **c** requiring anisotropic electronic treatment (e.g., for halogen bonding). Here, example of mitogen-activated protein kinase 1 complex is shown (3dy7^62^).

First, poses may be wrongly considered as failure due to ignored symmetry. In many cases, the poses were docked to a symmetric protein segment, for example, HIV protease with a binding site that is constituted by a homodimer with C2 symmetry. In many instances, the model will provide the two possible symmetric poses in the top 2-ranked list, but only one pose is provided in PDBBind (Fig. 5a, b)^32,33^.

Second, most of the failed systems contain top-ranked poses with RMSD values just above 2 Å (2.0–3.5 Å). Those RMSD deviations are largely due to the presence of solvent-exposed ligand moieties in alternative positions (Fig. 5c, d). These groups, however, display typically a high degree of flexibility and their exact position is often uncritical or irrelevant for protein-ligand binding. Previous studies have shown that a substantial deviation from the experimental structure due to such a solvent-exposed group can notably increase the RMSD even when the important binding features are conserved^34^. This was a major cause for wrong binding pose classification.

Third, several systems have crystallographic electron density maps with evidence for alternative, isoenergetic ligand poses in the binding site. Systems with potential alternative ligand poses that match the electron densities can be identified using the method qfit-ligand^35^. The fit of those alternative binding poses to the experimental electron density can be quantified using the real-space *R*-factor (RSR) by comparing the experimental electron density to the expected density for the alternative predicted ligand pose^34^. Using this analysis, several systems were identified where the top pose, although not in line with the original PDB structure, was consistent with the experimental electron density, for example, EphB4 kinase domain inhibitor complex (Fig. 5e, f). All those three reasons are reflected in ligand occupancy values smaller than one in the corresponding PDB structures. Analysis of all ligands from the PDB structures of our data set revealed that 2015 out of 14,021 ligands have documented occupancy values <1 (determined by the atom having the lowest occupancy value). The list of systems with low occupancy is provided as Supplementary Data 1. Whereas exclusion of all systems with lower-than-one occupancy may be one option to improve model performance, for drug-design applications it would be more beneficial to identify or predict those uncertain poses, for example, flexible tails with stable ligand core.

Fourth, some interactions, such as halogen-bonding, metal-ligand, or co-factor-ligand interactions (Fig. 5g, h), are comparably rare within the training set compared to standard interactions, such as hydrogen bonding, and so on. Furthermore, these interactions are often poorly modeled by atom-centered isotropic distribution functions. For example, halogen-bonding interactions require a modeling of anisotropic electron distributions to represent the *σ*-hole of halogen atoms^36,37^. The atom-centered density in the CNN input layers is incapable of treating those features. The CNN model, therefore, is incapable of learning these interaction features.

To correct for the symmetry issue, the symmetric binding mode from the original X-ray structure was included as additional native pose for comparison. Similarly, alternative poses consistent with the experimental electron density using RSR were considered as correct predictions.

To overcome the limitations of RMSD as the sole success criterion, in particular for solvent-exposed ligands, we made two changes to the loss function and performance evaluation. First, atoms with high solvent exposure are removed from the RMSD calculations (see Methods). Second, the contact mode score (CMS)^38^ (see Methods) was added as additional criterion to the loss function, measuring how many of the native contacts were conserved in a binding pose of consideration.

In order to better model interactions, such as halogen bonding, the extended electron distribution (XED)^39^ forcefield was utilized, which incorporates anisotropic atomic charge distributions (see Methods). To incorporate the anisotropic charge distribution in a form of 3D images compatible with the atom-type densities, first the interactions of a positive and negative probe with the ligand and protein were computed separately. The maxima of those interactions were identified. Additional input channels representing the interactions with the two probes were included in the model by placing Gaussian distribution functions on the centers of the extrema.

Using the general set from PDBBind, a model was trained on 12,694 protein-ligand systems^31^. The model performance on the test set was enhanced considerably to the previous models with 89% of native-like predictions in the top 1 (98% in top 3, 99% in top 5) (Fig. 4a, DeepWATsite(occ, large, RMSD + CS)). The majority of remaining false predictions can be attributed to low-affinity ligands, frequently fragment-sized molecules. Simulation and experimental studies^40–42^ for this class of ligands, however, revealed the likelihood of alternative binding modes.

Finally, model performance was analyzed separately for each of the 1036 protein families in training and test set. For statistical reasons, the analysis was focused on families with at least 20 different protein-ligand complexes in the data set. The final DeepWATsite model improves binding pose prediction for all of those families compared to standard scoring using Smina, highlighting the generalizability of the model to diverse protein targets (Supplementary Note 2).

To further test the generality of the CNN model, the full set of 14,740 compounds (12,694 training and 2046 test compounds) were combined and subsequently split into three diverse groups of approximately equal size (see Methods). Three-group cross-validation was performed on this set with almost equal prediction performance for the left out data. A native-like pose was identified at top-1 rank with 91%, 90%, and 88% for the three cross-validation models, respectively.

In this manuscript, only one classical scoring function was used for comparison. The reason for this selection are recent comparison studies on standard scoring functions that revealed overall convergence of those classical scoring functions in correctly predicting binding poses^43^. With respect to machine learning scoring functions, the same training and test set was applied on a variety of different machine learning architectures. A concise summary of methods and results of this study is found in Supplementary Note 3. None of those tested methods achieved the same accuracy in binding pose prediction as the final DeepWATsite model.

### Analysis of relevant features

While the pose ranking performance of DeepWATsite is significantly improved compared to standard protein-ligand-based CNN, it is critical to understand how the inclusion of hydration information caused the quality increase. Furthermore, the utility of DeepWATsite and CNN-based scoring in general as drug-design tools critically depends on the human interpretability of the model. Thus, we aimed to generate a detailed analysis method for interpreting the CNN models and for identifying critical features for protein-ligand binding. This could guide the rational optimization of molecules with a new level of accuracy and detail compared to standard docking approaches.

Despite impressive performances of CNNs in many applications related to image recognition, the models act typically as “black boxes” with a lack of interpretability. This “black-box” character is the result of the underlying nonlinear structure of the neural network. LRP is a recent method that aims to map the outcome of a model back to the input layers^44^. An adaptation of the initial method was recently tested on the study of protein-ligand interaction prediction^45^. The initial LRP, however, allowed for the mixture of positive and negative relevance values that results in artifacts such as large positive or negative values distant to the binding site.

In this paper, we implemented a new LRP methodology for interpreting generic multilayer neural networks based on the deep Taylor decomposition method^23^ by modifying a fork of Gnina^45,46^. Deep Taylor decomposition is a method to explain the individual predictions of deep neural networks in terms of the input layers. It functions by performing a layer-by-layer backward pass on the network starting from the classification between native (output value = 1) and decoy pose (output value = 0) using the following rules: According to the new LRP analysis, this positive defined classifier outcome in node *j* in output layer *N* (=classification as active pose for given input *x*), named relevance ${R}_{j}^{N}(x)$, needs to be conserved in each layer during the backward pass

1 $$\mathop {\sum}\limits_{i}{R}_{i}^{1}(x)=\mathop {\sum}\limits_{i}{R}_{i}^{2}(x)=\cdots =\mathop {\sum}\limits_{i}{R}_{i}^{N-1}(x)={R}_{j}^{N}(x).$$

Furthermore, the relevance in each layer measures the contribution of each node to the decision that the pose is native with zero meaning no contribution. Therefore, all relevance values should be positive or zero

2 $$\forall i,K,x:{R}_{i}^{K}(x)\ge 0.$$

The Taylor expansion at each node *i* of layer *N* − 1 estimates the contribution of this node to the positive outcome on node *j* (=native pose) in the output layer *N*. The relevance values from layer *N* − 1 are subsequently propagated to layer *N* − 2 using Taylor decomposition on each node *i* of layer *N* − 2 with respect to its overall relevance contribution of layer *N* − 1. Considering that the output value of layer *N* and the output of each ReLU node in the convolutational layers is non-negative, the following *z*^+^ rule can be derived for the relevance back-propagation^23^

3 $${R}_{i}^{K-1}(x)=\mathop {\sum}\limits_{j}\frac{{z}_{ij}^{+}}{{\sum }_{m}{z}_{mj}^{+}}{R}_{j}^{K,+}(x)$$

using only positive activation contributions ${z}_{ij}^{+}={x}_{i}{w}_{ij}^{+}$, that is,

4 $${w}_{ij}^{+}=\left\{\begin{array}{ll}{w}_{ij}&\,\,\,{\text{if}}\;{w}_{ij}\ge 0,\text{}\,\\ 0&\,{\text{if}}\;{w}_{ij} \, < \, 0\text{}\,\end{array}\right.$$

and propagating relevances only through positively activated nodes

5 $${R}_{j}^{K,+}(x)=\left\{\begin{array}{ll}{R}_{j}^{K}(x)&\,\,{\text{if}}\;\sum _{i}{x}_{i}{w}_{ij}+{b}_{j}\ge 0,\text{}\,\\ 0&\,{\text{if}}\;\sum _{i}{x}_{i}{w}_{ij}+{b}_{j} \, < \, 0\text{}\,,\end{array}\right.$$

where *x*_*i*_ is the activation of node *i* in layer *K* − 1, *w*_*ij*_ the weight of connection between node *i* in layer *K* − 1, *j* in layer *K,* and *b*_*j*_ the bias in layer *K*.

**Fig. 6:**
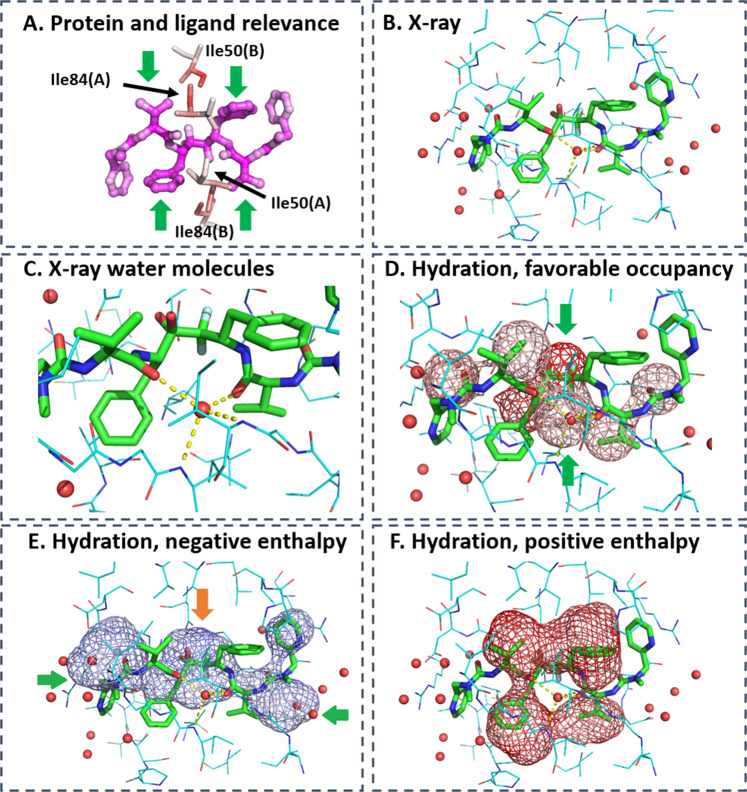
LRP analysis on HIV-1 protease in complex with inhibitor A79285. **a** Higher relevance is highlighted by darker red color for binding site residues (shown as sticks) and by darker magenta color for ligand atoms. **b** X-ray structure of complex of protein (lines, carbon in blue) and ligand (sticks, carbon in green) with surrounding water molecules (red spheres). PDB-code: 1dif^63^. **c** Water-mediated interaction between protein and ligand. **d** LRP analysis on water occupancy showing relevant water density, in particular around the center of the ligand (green arrows). The lower hydration density overlaps with the water molecule engaged in mediating interactions between ligand and protein. The upper hydration density coincides with a water molecule that is replaced by two hydroxyl groups forming critical and very stable interactions with aspartate side chains of the protein. **e** This particular water density was predicted with negative, that is, favorable enthalpy (orange arrow), in the LRP analysis focusing on negative enthalpy. First-shell water molecules with favorable enthalpy are additional relevant hydration features (green arrows). **f** Relevant features with positive enthalpy overlap with hydrophobic ligand moieties (isopropyl and phenyl groups) highlighting the importance of desolvation to ligand binding. Darker color means higher relevance in predicting native binding pose.

LRP analysis identifies the binding site residues and ligand groups critical for protein-ligand interactions in the native binding mode. It further highlights the water density most crucial for ligand binding. For example, Fig. 6a identifies the central region of the ligand to be the most important for its interaction with the protein. The side chains of residue Ile84 (chain B) and Ile50 (chain A) form critical hydrophobic contacts with the isopropyl and phenyl groups of the ligand (green arrow) anchoring the ligand in the binding site of the protein. The backbone amines of Ile50(A) and Ile50(B), which are predicted by the CNN model with high relevance, form critical water-mediated interactions with two hydrogen-bond acceptor groups of the ligand (Fig. 6b, c).

Co-localized with this central region are highly relevant water molecules (Fig. 6d) demonstrating that DeepWATsite identifies critical solvation contributions for protein-ligand interactions. One water molecule (lower green arrow) coincides with the water-mediating protein-ligand interactions. Another water molecule (upper green arrow) forms strong interactions with two aspartate residues in the unbound form of the protein. Even in the unbound state, WATsite predicted a favorable enthalpy value for this water location (Fig. 6e, orange arrow). This water molecule is replaced by a dihydroxy group of the ligand, strongly anchoring the ligand in the binding site of the protein. Additional solvent density with significant relevance and positive enthalpy overlaps with hydrophobic ligand groups, that is, the two isopropyl and phenyl groups of the symmetric ligand (Fig. 6f). As these water molecules are replaced upon ligand binding and therefore contribute favorably to the binding, they highlight the importance of desolvation as a significant driving force for protein-ligand association.

**Fig. 7:**
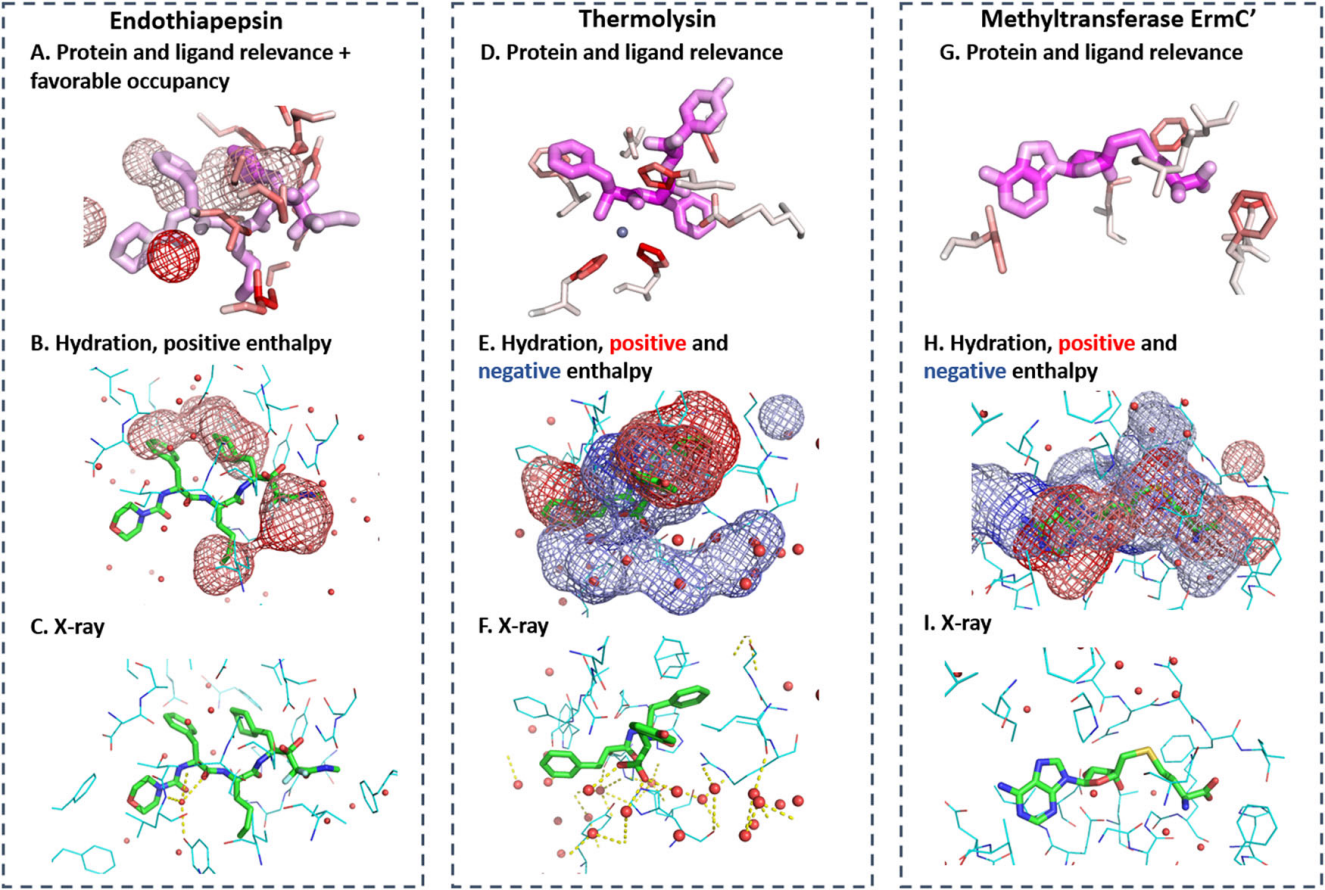
LRP analysis reveals different roles of solvation. **a**–**c** LRP analysis on endothiapepsin complexed with CP-81,282 (PDB-code: 1epo^64^) (**a**) showing relevant ligand atoms, protein residues, and water occupancy. **b** Water density with relevant positive desolvation enthalpy overlaps with hydrophobic groups of ligand highlighting the contribution of desolvation to the ligand binding to hydrophobic binding site moieties. **c** X-ray structure of protein-ligand complex highlighting the water-mediated interactions between protein and ligand (yellow dashed line) coinciding with the water occupancy of highest relevance. **d**–**f** LRP analysis on thermolysin complexed with (2-sulphanyl-3-phenylpropanoyl)-Phe-Tyr (PDB-code: 1qf0^65^) highlighting **d** most relevant protein residues and ligand atoms, and **e** relevant positive (red) and negative (blue) enthalpy contributions of hydration. **f** X-ray structure of protein-ligand complex highlighting the extended first-layer water network around the solvent-exposed ligand moiety. **g**–**i** LRP analysis on rRNA methyltransferase ErmC′ bound with S-adenosyl-l-homocysteine (PDB-code: 1qan^66^) highlighting **g** most relevant protein residues and ligand atoms, and **h** relevant positive (red) and negative (blue) enthalpy contributions of (de)solvation. **i** X-ray structure of protein-ligand complex. Same representations as in Fig. 6.

The LRP analysis of all studied targets identified relevant water density for the correct prediction of protein-ligand complexes, highlighting the importance of desolvation of free-energy contributions and water-mediated interactions for protein-ligand association. For example, in the complex of CP-81,282 with endothiapepsin, a water molecule mediating critical interactions between protein and ligand (Fig. 7c) is identified with the highest relevance (Fig. 7a, dark red density). Water density with positive enthalpy, that is, unfavorable water locations, are identified in hydrophobic moieties with high relevance (Fig. 7b). Those regions overlap with hydrophobic ligand moieties. This again underlines the importance of desolvation as a driving force for ligand binding, also commonly termed the hydrophobic effect of protein-ligand association.

In thermolysin complexed with (2-sulfanyl-3-phenylpropanoyl)-Phe-Tyr, the LRP analysis reveals that the coordination of the ligand to the zinc ion stabilized by three histidine residues is essential for ligand binding (Fig. 7d). Relevant water density overlapping with the ligand is identified, highlighting again desolvation of free-energy contributions to binding. In addition, water density with high relevance is identified in an extended region adjacent to the ligand (blue density in the lower part of Fig. 7e). This density co-localizes with a network of x-ray water molecules. Most of those water molecules do not mediate interactions between protein and ligand and none is replaced by the ligand upon association. These water molecules instead form a stable network of first-shell water molecules surrounding the exposed phenyl moiety of the ligand. The stability of this network is documented by the negative desolvation enthalpy.

Using a combination of X-ray crystallography, ITC measurements and simulations, Klebe and co-workers^24^ recently studied this particular water network around thermolysin inhibitors. They were able to interpret surprising thermodynamic structure–activity relationships with the stability or partial rupture of this network cage around the solvent-exposed ligand moieties. The DeepWATsite CNN model identifies the importance of this water network without any specific user input besides binding pose and WATsite information.

In addition to thermolysin, the DeepWatsite model was able to identify other protein systems with important first-shell water layers around exposed ligand moieties. This observation suggests that this role of water molecules may be of general importance and currently under-appreciated compared to desolvation effects and water-mediated interactions. For example, in HIV protease with bound A79285, water density with negative enthalpy was identified around the ligand (Fig. 6e, green arrows). In ribosomal RNA methyltransferase ErmC′ bound with S-adenosyl-l-homocysteine, water density with negative enthalpy and high relevance is identified around the sulfur-containing moiety of the ligand (upper part of Fig. 7h) co-localized with four x-ray water molecules. Those water molecules neither directly mediate interactions between protein and ligand nor are replaced by the ligand upon association.

**Fig. 8:**
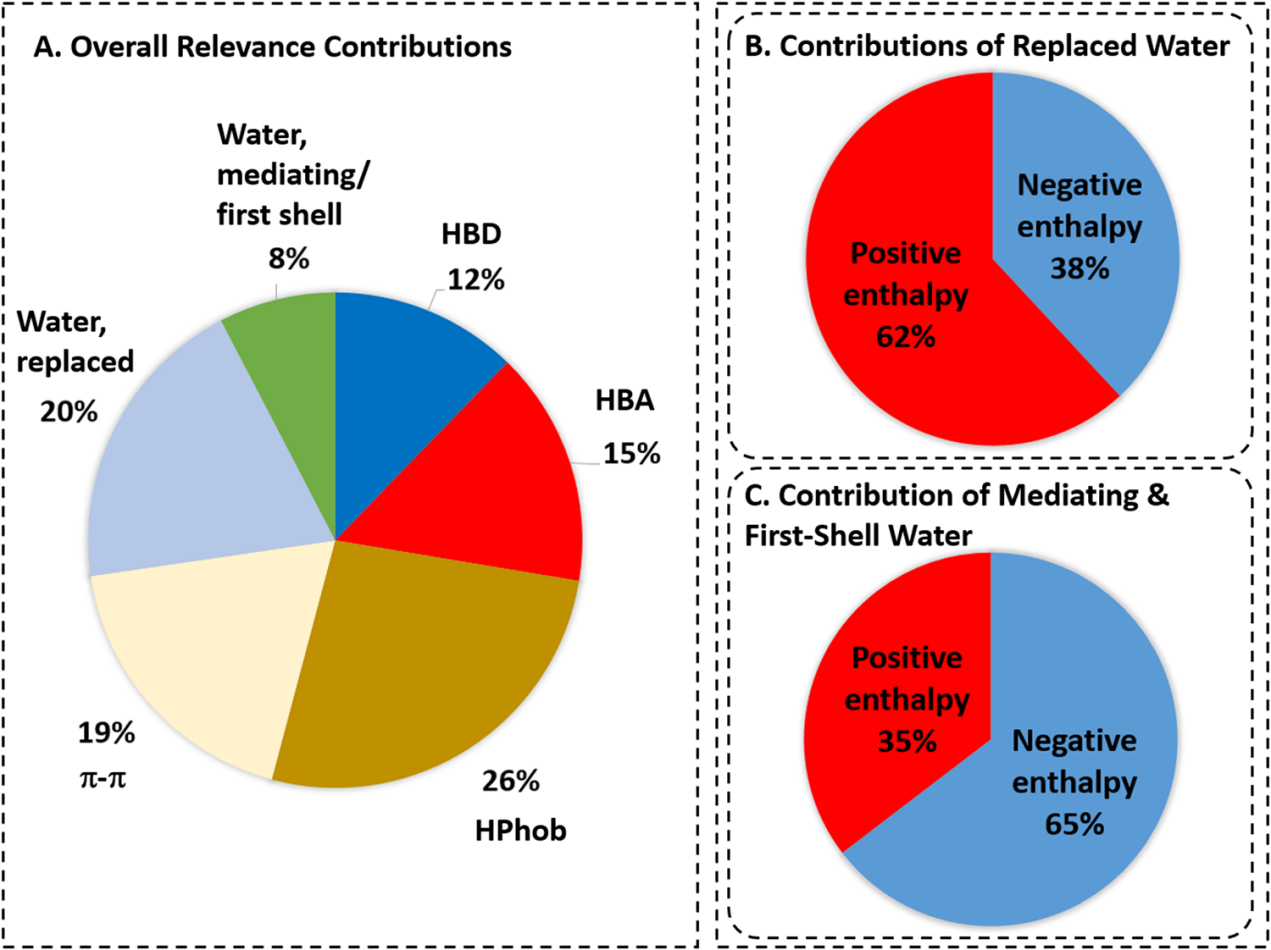
Relevance contributions of CNN score. **a** Average relative contribution of different interaction and hydration types to predicted CNN score of native pose. **b** Contribution of water molecules, which are replaced by ligand binding, with different enthalpy footprint to CNN score. The water molecules have predominantly positive enthalpy values, significantly contributing to the binding affinity as they are released to bulk solvent upon ligand binding. **c** Contribution of water molecules, which mediate protein-ligand interactions or form first-shell water layers around protein-ligand surface, with different enthalpy footprint to CNN score. The water molecules have predominantly negative enthalpy values, significantly contributing to the binding affinity as they adapt energetically favorable state in protein-ligand complex.

To analyze the overall importance of the different input features, we quantified the average relevance of ligand atoms, protein atoms, and water density over all 2046 test systems for the prediction of the native ligand pose. Figure 8a displays the average contributions of the different elements of protein-ligand binding to the overall relevance. Whereas protein and ligand atoms involved in hydrophobic and hydrogen-bond interactions are the dominant contributors to the overall relevance (26% and 27%, respectively), water molecules that are replaced upon ligand binding (20%) significantly contribute to the relevance as well. While the average contribution to the overall relevance of water-mediated interactions and first-shell water layers with 8% is comparably smaller, there are 130 systems where these features contribute more than 20% to the total relevance; in 21 systems the contribution is even more than 30%. Analyzing the enthalpy of water molecules relevant for ligand binding (Fig. 8b), we identified that the majority of the water molecules that are replaced by the ligand are enthalpically unfavorable compared to bulk water and therefore contribute favorably to the binding affinity upon replacement. In contrast, the majority of water molecules mediating protein-ligand interactions or forming hydration shells around the ligand (Fig. 8c) have on average a more negative enthalpy compared to the bulk state, consistent with role in stabilizing protein-ligand interactions.

In conclusion, deep learning has established itself as one of the leading methodologies in image and speech recognition, but has only recently entered the field of protein-ligand modeling. Also recent is the emerging emphasis on the importance of inclusion of (de)solvation in modeling protein-ligand interactions. In this article, we presented the first deep-learning approach to model protein-ligand interactions incorporating hydration information. The significant improvement of DeepWATsite for pose ranking compared to standard CNN models or empirical scoring functions highlights the utility of this approach for a more precise modeling of protein-ligand interactions.

The drastic reduction in failure rate to predict the correct binding pose at top rank from around 40% in standard docking using Vina to around 10% using the optimized DeepWATsite will have broad impact to the drug-design process. Even without experimental protein-ligand complex structure, rational structure-based lead optimization will be made possible without the significant ambiguity of current docking-based approaches. Furthermore, the predicted pose can be directly used for subsequent free-energy calculations without the uncertainty of considering several alternative docking poses. Thus, the additional WATsite simulation (which only takes about half an hour on today’s GPU processors) is more than well invested considering the simulation lengths of free-energy calculations.

It should be noted that the method described here is a re-scoring method, that is, docking is carried out initially with Smina and poses are re-scored using the CNN, which take into consideration the solvation grids. Ligand flexibility is thus fully included on a dihedral level as part of the sampling part of Smina docking. As it is common practice in scoring-docking evaluations, the current study uses re-docking into the fixed protein structure. This is consistent with the fact that the water grids are computed using MD simulations with restrained protein to ensure statistical stability of the thermodynamic values. In the future, we plan to incorporate protein flexibility by generating grids for an ensemble of different protein conformations as discussed in a recent publication by our group^47^.

Of similar importance as the pose ranking quality is the new level of interpretability of the underlying CNN models using the LRP analysis in our study. Several selected examples highlight that the model and its analysis can identify residues and ligand functional groups critical for protein-ligand binding. Furthermore, different types of hydration effects essential for ligand binding were identified, including desolvation, water-mediated interactions, and the formation of stable networks of water molecules around exposed ligand moieties.

This information can be valuable in various contexts. For example, the identification of important residues and ligand atoms for interaction can guide the rational modification of a ligand without relying on an imprecise energetic analysis based on simplistic scoring functions that neglects the environmental context of interactions. The identification of critical water-mediated interactions can guide the selection of water molecules to be included during ligand design or during docking. The analysis is also able to reveal enthalpically favorable water networks on the boundary of binding sites that are currently neglected by most drug-design projects, but can be important for the observed structure–activity relationships as recently highlighted.^24,25^ Our analysis reveals that this ignored facet of solvation is more widespread in protein-ligand complexes than currently recognized.

## Methods

### Datasets information

For validating and benchmarking the accelerated WATsite version, HIV-1 protease (PDB: 1PHX) was used as protein system. In addition to HIV-1 protease, dihydrofolate reductase was used to measure the efficiency of the new implementation. This system allows for direct comparison with other MD programs such as Amber, Charmm, or OpenMM, where it has been used as standard benchmark.

For training and validation of two different sets of CNN models 2423 and 14,740 protein-ligand structures from PDBbind were used, respectively (see Supplementary Data 2). For each protein, the co-crystallized ligand was removed from its complex structure in order to profile the hydration site information in the binding site of each protein. Protein structures were prepared using Schrodinger’s Protein Preparation Wizard^48^. In short, hydrogen positions, bond orders, and protonation states of histidines, glutamic, and aspartic acids, and side-chain conformations of asparagine and glutamine side chains were optimized using the default protocol.

### Accelerated WATsite implementation

To make the inclusion of WATsite hydration data into CNN possible, hydration profiling had to be performed for a large set of protein structures. This required a new implementation of WATsite with significant speed-up.

WATsite simulations were performed using OpenMM on GPU architecture, where the OpenMM code was modified to allow for on-the-fly calculation and report of interaction energies between each water molecule and its surrounding. For each water molecule in the system, the interaction energy between this water molecule and all other protein, co-factor, and water residues is calculated asynchronously to the MD propagation increasing the speed of the simulation. The water-interaction energies are subsequently averaged over all MD snapshots to calculate the desolvation enthalpy in WATsite. Desolvation entropy is computed based on the MD trajectory as described in previous publications^13,14^. For further speed-up of the MD simulation protocol, the protein was truncated beyond a sphere positioned in the center of the binding site. This truncation is motivated by the fact that the protein is restrained in all WATsite simulations to achieve convergence in water-occupancy and free-energy profiling.

The latest WATsite version including WATsite App for easy visualization and simulation setup can be downloaded from https://hub.docker.com/r/lilllab/watsite3.

### MD simulations

See Supplementary Methods for details.

### Hydration site analysis

A detailed description of the WATsite methodology can be found in previous publications^13,14^. In short, a 3D grid was placed over the user-defined binding site. The occupancy of water molecules was distributed onto the 3D grid using all snapshots generated throughout the production run of the MD simulation. The distribution of the occupancy was then normalized and QT clustering algorithm was used to identify the pronounced peaks that define the hydration site locations. For each identified hydration site, WATsite tools were used to compute the enthalpic (ΔH_hs_) and entropic (ΔS_hs_) change of transferring a water molecule from the bulk solvent to the hydration site of the protein binding site. The output files from the modified OpenMM-WATsite code containing the individual water-interaction energies were used in this step.

### Grid-based hydration analysis

The previous implementations of WATsite predicted hydration sites representing the cluster centers of high solvent density regions (see previous section). This localized representation of water occupancy is not well suited as input for CNN, which requires 3D grid data. Inspired by GIST^21^, an alternative grid-based analysis was implemented into WATsite. Similar to hydration site prediction, a 3D grid was placed over the user-defined binding site. The occupancy of water molecules was distributed onto the 3D grid using all snapshots generated throughout the production run of the MD simulation. In contrast to standard WATsite, the occupancy is not clustered into hydration sites, but every grid point with larger than twice the bulk occupancy is considered as a “pseudo-hydration site” and any water molecule within 1 Å radius throughout the MD trajectory is considered to contribute to it. Only the oxygen atom of a water molecule is considered as criterion for inclusion. The desolvation enthalpy and entropy of the “pseudo-hydration site” is calculated similarly as in the original hydration site analysis using the contribution of any water molecule within 1 Å radius throughout the MD trajectory.

In order to achieve fast convergence of the thermodynamic profile of hydration data, in particular for water molecules in enclosed binding sites, we utilized our recently developed protocol that uses GAsol, which relies on 3D-RISM, for initial water placement^26^.

### Convergence of grid-based energy analysis

See Supplementary Methods for details.

### System truncation

For speed-up of the MD simulation protocol, the protein was truncated beyond a threshold radius around any ligand atom. Different threshold radii or cutoffs (12, 15, 17, 20, 25, and 30 Å) were tested. We analyzed the impact of such truncations by comparing the WATsite predictions to those of the full protein system. For all truncated systems, a python script is used to add capping acetyl (ACE) and amide (NME) groups to the break points in the protein sequence. The protein structures were then solvated in an orthorhombic box of water molecules. A minimum distance of 10 Å between any protein atom and the faces of the box was chosen. Hydration site and grid-based analysis were performed using 20 ns MD simulations of HIV-1 protease truncated at different cutoff distances around the center of the binding site. For the grid-based analysis, the values of the overlap coefficient (OC) (Eq. (6)).

6 $${\rm{OC}}=\sum\limits _{i=1}^{N}\,\min\left(\frac{p_{i}^{1}}{\sum _{j=1}^{N}{p}_{j}^{1}};\,\frac{p_{i}^{2}}{\sum _{j=1}^{N}{p}_{j}^{2}}\right)$$

of energy grids comparing truncated (energy values on grid point *i*${p}_{i}^{1}$) with full system (${p}_{i}^{2}$) are reported in Supplementary Table 3. With OC value of 0.91, 0.91, and 0.93, truncation at 12 Å seems to be able to reproduce the results of the full system with an additional 80 ns per day speed increase for the test system. It should be noted that the full HIV-1 protease system consists of only 198 protein residues. Thus, the speed increase is not as significant as expected, in particular in the range 17–25 Å truncation. The advantage of truncation is more pronounced for larger systems. The system with a 30 Å cutoff truncation consists of the entire protein and was used as reference for the full system. To be on the safe side, we selected a truncation radius of 15 Å for the current study.

### Efficiency measures of accelerated WATsite3.0 implementation

See Supplementary Methods for details.

### Binding pose generation

Protein–ligand complexes were randomly selected from the PDBbind database for pose prediction with DeepWATsite. Similar to the work of Ragoza et al.^5^, the co-crystalized ligand was extracted and re-docked using Smina^49^ with the AutoDock Vina scoring function^50^. Docking was performed into rigid protein structures with all water molecules stripped. Protonation states for ligand and protein were determined using OpenBabel^51^. The “autobox_ligand” and “autobox_add” switches were used to define a docking box that extends the dimensions of the co-crystalized ligand by 12 Å in all directions. Exhaustiveness of 40 (default: 8) was used for more extensive sampling of poses. Twenty-five poses for each system were generated and classified based on their RMSD to the X-ray binding pose. Poses with an RMSD  <2 Å are considered native poses, other poses as decoy poses. As the focus is on the development of an improved scoring function, only systems with at least one native-like pose (RMSD  <2 Å to native binding pose) were selected for subsequent CNN modeling.

### Inclusion of hydration information into CNN scoring

Protein and ligand atoms were modeled as density on a 3D grid encompassing the binding site of the protein. These data were provided to the CNN model in the form of 34 different 3D grids as input channels representing the density of 34 distinct atom types (16 protein and 18 ligand types). The atom-type classification was based on Smina atom-type rules^49^. The density distribution of each protein or ligand grid was computed using the following piece-wise continuous function^5^

7 $${\rho }_{i}(r)=\left\{\begin{array}{ll}\exp (-\frac{2{r}^{2}}{{R}_{i}^{2}})&\,{\text{if}}\;0 \, \le \, r \, < \, {R}_{i},\text{}\,\\ \frac{4}{{e}^{2}{R}_{i}^{2}}{r}^{2}-\frac{12}{{e}^{2}{R}_{i}}r+\frac{9}{{e}^{2}}&\,\hskip 15pt{\text{if}}\;{R}_{i} \, \le r \, < \, 1.5{R}_{i},\text{}\,\\ 0&\,\hskip -3pt{\text{if}}\;1.5{R}_{i}\le r,\text{}\,\end{array}\right.$$

where *R*_*i*_ is the van der Waals radius of atom-type *i* and *r* is the distance between atom center and grid point at which the atom density was computed. Each 3D grid covered a volume of 24 × 24 × 24 Å^3^ using a grid spacing of 0.5 Å. The data were provided as MolGrid input layers to Gnina^5^ with Caffe as the deep-learning framework^52^.

Occupancy and thermodynamic values generated by WATsite on 3D grids encompassing the binding site were provided as additional input channels to the CNN model. We developed two different CNN models including hydration information. One model was constructed using only hydration occupancy in addition to protein and ligand density. This model is purely based on density information of protein, ligand, and water molecules.

The other model, in contrast, utilizes energetic values of hydration in addition to protein and ligand density. Only enthalpy and entropy were used as input, as the free energy is the sum of the two. To allow for normalized input between 0 and 1, a hyperbolic tangent function was used to scale the thermodynamic values. Whereas the entropy values were always positive with respect to bulk solvent entropy, the enthalpy values can assume positive and negative values. Thus, we separated the enthalpy data into two different input channels, one for positive enthalpy values and one for negative values. The latter were taken as absolute values before normalization by the hyperbolic tangent function.

Thus, in total 35 and 37 input channels are provided to the two CNN models in the form of 3D grids, respectively. CNN models using only the 34 protein and ligand input channels were generated for comparison, to validate the importance of solvation information in the CNN modeling.

In addition to the input layer, the CNN architecture contained three convolutional layers with rectified linear unit (ReLU) activations coupled with intermittent max pooling layers, one fully connected layer and a final softmax layer, which mapped a probability for the two output options of the classifier model, native (RMSD to x-ray pose  <2 Å) or decoy pose (Supplementary Fig. 2).

The models were trained using the Caffe framework for 40,000 iterations using a stochastic gradient descent variant. In the first model, only 377 randomly selected protein structures were used for training. This choice guarantees the rigorous applicability validation of the model on a randomly selected, large and diverse external test set of 2046 structures, comprising 369 different protein families based on PFAM classification (Supplementary Table 1).

To allow for sufficient optimization of the model, the training set was augmented using random rotation and translation (up to 2 Å) of the input images (3D grids). In a second model, the training set was expanded to 12,694 systems from the general set of PDBBind^31^.

### Validation study using multi-fold cross-validation

After verifying that the model can predicted the binding poses for a diverse set of protein families, we decided to use even more challenging and robust tests in form of cross-validation. Cross-validation was performed similar to Ragoza et al^5^. A distance matrix was computed between the proteins according to their sequence and ligand similarity. The latter was computed between ligands according to their SMILES fingerprints^53^. Clustering was done for all systems where a system is added to a group if the sequence distance is <0.5 or ligand similarity is >0.9. Three balanced groups (=folds) were generated where large groups were iteratively assigned to the fold with fewest systems. The PDB ids for the different folds are provided in the accompanying file Supplementary_Data_2.txt.

### Alternative measures for pose prediction success

Due to its simplicity and ease of implementation, the RMSD criterion for binding mode prediction is typically used to discuss docking methods and applications. However, the use of RMSD as sole success criterion can be problematic. For example, small or highly symmetrical ligands are tending to have low RMSD values even if randomly placed in a small active site. On the contrary, large RMSD values are more frequently observed for large, flexible ligands even if most protein-ligand contacts are conserved. These large RMSD values are often caused by solvent-exposed moieties that are not important for protein-ligand binding. To overcome these limitations of RMSD as criterion for docking success, several alternative methods have been postulated for pose evaluations, such as relative displacement error^54^, interaction-based accuracy classification^55^, RSR^34^, and GARD (generally applicable replacement for rmsD)^56^. In this manuscript, we decided to use, in addition to standard RMSD, a modified RMSD excluding solvent-exposed groups and the CMS^38^ to measure the accuracy of docking poses.

*RMSD modified by solvent exposure*: A modified RMSD calculation was designed, in which portions of the ligands that are highly exposed to the solvent are ignored in the calculation of RMSD. To compute the modified RMSD, the ligand is split into a set of rigid fragments (Supplementary Fig. 3). The solvent-accessible surface area (SASA) of the fragments that comprise the molecule are computed by, first, determining the atomic SASA in the context of the bound protein using a Schrodinger script^57^. The SASA of each fragment is subsequently computed by summing up the SASA of each atom of this fragment. The ratio between the SASA of the fragment within the protein context and the SASA of the pose while removing the protein context (unbound ligand) was computed. Fragments with SASA ratio >0.25 are considered solvent exposed and were excluded from the RMSD computation. Using Schrodinger RMSD calculation tool, the ASL query language was utilized to compute the modified RMSD, excluding the solvent-exposed fragment. The modified RMSD was used to classify the poses either as near native or as decoy.

*Contact mode score*: RMSD (and the modified RMSD) is used as classification measure between native-like and decoy poses, but is not useful in a regression model to quantify remaining native contacts. Thus, poses with a significant number of native contacts but >2 Å RMSD enforce low CNN scores similar to poses without any native contacts.

To increase the robustness of the model by positively considering native interactions during CNN model training, CMS is used in addition to RMSD. CMS reflects the similarity of the protein-ligand interactions of a docking pose relative to the native structure (Supplementary Fig. 4)^38^. CMS ranges between 0 and 1 and can be intuitively used as a loss function for a regression model, here in the form of the pseudo-Huber loss function. Based on general observations, we consider a pose to be incorrect if the CMS value is <0.44^38^. Those low CMS values might reflect a pose without significant overlap with the native pose. Those non-overlapping poses should therefore not be used in the quantification of the quality of the interactions. In our case, we considered those poses as “undefined” and a hinge loss is used so that the contact score prediction loss is only incurred on the low-contact score poses if the contact score is predicted to be higher than the cutoff value of 0.44.

### Electrostatic interactions

To model interactions, such as halogen bonding, which require representation of anisotropic electron distributions, the XED forcefield was chosen^39^. XED provides a practical method to account for anisotropic and directional electrostatic interactions, such as halogen bonding or hydrogen bonding. In contrast to ab initio methods, it avoids prohibitive computational costs and difficulties in calculation setup for large molecules. To include XEDs compatible with the other input layers, which use Gaussian density functions, the interactions of a positive and a negative probe with the ligand are computed, and the maxima of those interactions are determined (Supplementary Fig. 5). Both the interactions with the protein and the ligand in a given pose were separately computed. Additional channels representing the interactions with the probes were included in the model using Gaussian distributions functions centered on the maxima of the interactions. The Gaussian width was chosen to be dependent on the magnitude of the interaction values at a given maximum.

## Supplementary information

Supplementary Information

Description of Additional Supplementary Files

Supplementary Data 1

Supplementary Data 2

Supplementary Data 3

Supplementary Data 4

## Data Availability

All data necessary to reproduce the data presented in this article are included in the manuscript or the supporting information. Other data supporting the findings of this study are available as Supplementary Data 1–4. A separate file describes the contents of the files.

## Code availability

Software and examples for WATsite and LRP analysis including CNN models are provided in the form of docker images. These images can be downloaded at https://hub.docker.com/r/lilllab/watsite3 and https://hub.docker.com/r/lilllab/deepwatsite_lrp, respectively.

## References

[1] Goh, G. B., Hodas, N. O. & Vishnu, A. Deep learning for computational chemistry. *J. Comput. Chem.* **38**, 1291–1307 (2017). 10.1002/jcc.24764 28272810

[2] Skalic M, Martíez-Rosell G, Jiménez J, De Fabritiis G (2018). PlayMolecule BindScope: large scale CNN-based virtual screening on the web. Bioinformatics.

[3] Xu Y, Chen P, Lin X, Yao H, Lin K (2019). Discovery of cdk4 inhibitors by convolutional neural networks. Fut. Med. Chem..

[4] Xavier MM (2016). SAnDReS a computational tool for statistical analysis of docking results and development of scoring functions. Comb. Chem. High Throughput Screen..

[5] Ragoza M, Hochuli J, Idrobo E, Sunseri J, Koes DR (2017). Protein–ligand scoring with convolutional neural networks. J. Chem. Inf. Model..

[6] Wallach, I. Dzamba, M. & Heifets, A. AtomNet: a deep convolutional neural network for bioactivity prediction in structure-based drug discovery. https://arxiv.org/abs/1510.02855 1–11 (2015).

[7] Ladbury JE (1996). Just add water! the effect of water on the specificity of protein–ligand binding sites and its potential application to drug design. Chem. Biol..

[8] Abel R, Young T, Farid R, Berne BJ, Friesner RA (2008). Role of the active-site solvent in the thermodynamics of factor xa ligand binding. J. Am. Chem. Soc..

[9] Böhm H-J (1994). The development of a simple empirical scoring function to estimate the binding constant for a protein-ligand complex of known three-dimensional structure. J. Comput. Aided Mol. Des..

[10] Eldridge MD, Murray CW, Auton TR, Paolini GV, Mee RP (1997). Empirical scoring functions: I. the development of a fast empirical scoring function to estimate the binding affinity of ligands in receptor complexes. J. Comput. Aided Mol. Des..

[11] Morris GM (1998). Automated docking using a lamarckian genetic algorithm and an empirical binding free energy function. J. Comput. Chem..

[12] Huang SY, Zou X (2010). Inclusion of solvation and entropy in the knowledge-based scoring function for protein–ligand interactions. J. Chem. Inf. Model..

[13] Hu B, Lill MA (2014). Watsite: Hydration site prediction program with pymol interface. J. Comput. Chem..

[14] Yang, Y., Hu, B. & Lill, M. A. In (Daisuke Kihara ed.) *Methods in Molecular Biology* 123–134 (Springer, New York, 2017). 10.1007/978-1-4939-7015-5_10 28451976

[15] Nittinger E (2018). Placement of water molecules in protein structures: from large-scale evaluations to single-case examples. J. Chem. Inf. Model..

[16] Young T, Abel R, Kim B, Berne BJ, Friesner RA (2007). Motifs for molecular recognition exploiting hydrophobic enclosure in protein–ligand binding. Proc. Natl Acad. Sci. USA.

[17] Higgs C, Beuming T, Sherman W (2010). Hydration site thermodynamics explain sars for triazolylpurines analogues binding to the a2a receptor. ACS Med. Chem. Lett..

[18] Abel R (2011). Contribution of explicit solvent effects to the binding affinity of small-molecule inhibitors in blood coagulation factor serine proteases. ChemMedChem.

[19] Lazaridis T (1998). Inhomogeneous fluid approach to solvation thermodynamics. 1. theory. J. Phys. Chem. B.

[20] Lazaridis T (1998). Inhomogeneous fluid approach to solvation thermodynamics. 2. applications to simple fluids. J. Phys. Chem. B.

[21] Nguyen CN, Young TK, Gilson MK (2012). Grid inhomogeneous solvation theory: hydration structure and thermodynamics of the miniature receptor cucurbit[7]uril. J. Chem. Phys..

[22] Balius TE (2017). Testing inhomogeneous solvation theory in structure-based ligand discovery. Proc. Natl Acad. Sci. USA.

[23] Montavon G, Lapuschkin S, Binder A, Samek W, Müller KR (2017). Explaining nonlinear classification decisions with deep Taylor decomposition. Pattern Recognit..

[24] Biela A, Betz M, Heine A, Klebe G (2012). Water makes the difference: Rearrangement of water solvation layer triggers non-additivity of functional group contributions in protein-ligand binding. ChemMedChem.

[25] Krimmer SG, Betz M, Heine A, Klebe G (2014). Methyl, ethyl, propyl, butyl: futile but not for water, as the correlation of structure and thermodynamic signature shows in a congeneric series of thermolysin inhibitors. ChemMedChem.

[26] Masters MR, Mahmoud AH, Yang Y, Lill MA (2018). Efficient and accurate hydration site profiling for enclosed binding sites. J. Chem. Inf. Model..

[27] Kovalenko A, Hirata F (1998). Three-dimensional density profiles of water in contact with a solute of arbitrary shape: a rism approach. Chem. Phys. Lett..

[28] Sindhikara DJ, Yoshida N, Hirata F (2012). Placevent: an algorithm for prediction of explicit solvent atom distribution-application to hiv-1 protease and f-atp synthase. J. Comput. Chem..

[29] Sindhikara DJ, Hirata F (2013). Analysis of biomolecular solvation sites by 3d-RISM theory. J. Phys. Chem. B.

[30] Fusani L, Wall I, Palmer D, Cortes A (2018). Optimal water networks in protein cavities with GAsol and 3d-RISM. Bioinformatics.

[31] Li H, Leung KS, Wong MH, Ballester PJ (2015). Low-quality structural and interaction data improves binding affinity prediction via random forest. Molecules.

[32] Bitencourt-Ferreira, G. & de Azevedo, W. F. In (Walter Filgueira de Azevedo Jr. ed.) *Methods in Molecular Biology* 251–273 (Springer, New York, 2019). 10.1007/978-1-4939-9752-7_16 31452110

[33] Pintro VO, de Azevedo WF (2018). Optimized virtual screening workflow: towards target-based polynomial scoring functions for HIV-1 protease. Comb. Chem. High Throughput Screen..

[34] Yusuf D, Davis AM, Kleywegt GJ, Schmitt S (2008). An alternative method for the evaluation of docking performance: Rsr vs rmsd. J. Chem. Inf. Model..

[35] van Zundert GCP (2018). qfit-ligand reveals widespread conformational heterogeneity of drug-like molecules in x-ray electron density maps. J. Med. Chem..

[36] Hernandes M, Cavalcanti SM, Moreira DR, de Azevedo W, Leite AC (2010). Halogen atoms in the modern medicinal chemistry: hints for the drug design. Curr. Drug Targets.

[37] Ford MC, Ho PS (2015). Computational tools to model halogen bonds in medicinal chemistry. J. Med. Chem..

[38] Ding Y (2016). Assessing the similarity of ligand binding conformations with the contact mode score. Comput. Biol. Chem..

[39] Bauer MR, Mackey MD (2019). Electrostatic complementarity as a fast and effective tool to optimize binding and selectivity of protein–ligand complexes. J. Med. Chem..

[40] Mpamhanga CP (2009). One scaffold, three binding modes: novel and selective pteridine reductase 1 inhibitors derived from fragment hits discovered by virtual screening. J. Med. Chem..

[41] Nair PC, Malde AK, Drinkwater N, Mark AE (2012). Missing fragments: detecting cooperative binding in fragment-based drug design. ACS Med. Chem. Lett..

[42] Hubbard RE, Chen I, Davis B (2007). Informatics and modeling challenges in fragment-based drug discovery. Curr. Opin. Drug Discov. Dev..

[43] Wang Z (2016). Comprehensive evaluation of ten docking programs on a diverse set of protein-ligand complexes: the prediction accuracy of sampling power and scoring power. Phys. Chem. Chem. Phys..

[44] Bach S (2015). On pixel-wise explanations for non-linear classifier decisions by layer-wise relevance propagation. PLoS ONE.

[45] Hochuli J, Helbling A, Skaist T, Ragoza M, Koes DR (2018). Visualizing convolutional neural network protein-ligand scoring. J. Mol. Graph. Model..

[46] Gnina github. https://github.com/gnina/gnina. Accessed 15 October 2019.

[47] Yang Y, Lill MA (2016). Dissecting the influence of protein flexibility on the location and thermodynamic profile of explicit water molecules in protein–ligand binding. J. Chem. Theory Comput..

[48] Madhavi Sastry G, Adzhigirey M, Day T, Annabhimoju R, Sherman W (2013). Protein and ligand preparation: parameters, protocols, and influence on virtual screening enrichments. J. Comput. Aided Mol. Des..

[49] Koes DR, Baumgartner MP, Camacho CJ (2013). Lessons learned in empirical scoring with smina from the csar 2011 benchmarking exercise. J. Chem. Inf. Model..

[50] Trott, O. & Olson, A. J. Autodock vina: improving the speed and accuracy of docking with a new scoring function, efficient optimization, and multithreading. *J. Comput. Chem*.**31**, 455–461 (2010). 10.1002/jcc.21334 PMC3041641 19499576

[51] O’Boyle NM (2011). Open babel: an open chemical toolbox. J. Cheminform..

[52] Jia, Y. et al. Caffe: convolutional architecture for fast feature embedding. In *Proc. 22nd ACM International Conference on Multimedia*, MM’14, 675–678 (ACM, New York, 2014).

[53] Schwartz J, Awale M, Reymond J-L (2013). Smifp (smiles fingerprint) chemical space for virtual screening and visualization of large databases of organic molecules. J. Chem. Inf. Model..

[54] Abagyan RA, Totrov MM (1997). Contact area difference (cad): a robust measure to evaluate accuracy of protein models. J. Mol. Biol..

[55] Kroemer RT (2004). Assessment of docking poses: interactions-based accuracy classification (ibac) versus crystal structure deviations. J. Chem. Inform. Comput. Sci..

[56] Baber JC, Thompson DC, Cross JB, Humblet C (2009). Gard: a generally applicable replacement for rmsd. J. Chem. Inf. Model..

[57] Script atomic_sasa.py. https://www.schrodinger.com/scriptcenter. Accessed 15 October 2019.

[58] Kawasaki Y (2010). How much binding affinity can be gained by filling a cavity?. Chem. Biol. Drug Des..

[59] Yan X (1997). Structure-based identification of a ricin inhibitor. J. Mol. Biol..

[60] Groves MR, Yao Z-J, Roller PP, Burke TR, Barford D (1998). Structural basis for inhibition of the protein tyrosine phosphatase 1B by phosphotyrosine peptide mimetics. Biochemistry.

[61] Bardelle C (2008). Inhibitors of the tyrosine-kinase EphB4. Part 2: Structure-based discovery and optimisation of 3, 5-bis substituted anilinopyrimidines. Bioorg. Med. Chem. Lett..

[62] Tecle H (2009). Beyond the MEK-pocket: can current MEK kinase inhibitors be utilized to synthesize novel type III NCKIs? Does the MEK-pocket exist in kinases other than MEK?. Bioorg. Med. Chem. Lett..

[63] Silva AM, Cachau RE, Sham HL, Erickson JW (1996). Inhibition and catalytic mechanism of HIV-1 aspartic protease. J. Mol. Biol..

[64] Veerapandian B (2008). Direct observation by X-ray analysis of the tetrahedral “intermediate” of aspartic proteinases. Protein Sci..

[65] Gaucher JF (1999). Crystal structures of *α*-mercaptoacyldipeptides in the thermolysin active site: structural parameters for a Zn monodentation or bidentation in metalloendopeptidases. Biochemistry.

[66] Schluckebier, G., Zhong, P., Stewart, K. D., Kavanaugh, T. J. & Abad-Zapatero, C. The 2.2 Å structure of the rRNA methyltransferase ErmC$$^{\prime}$$ and its complexes with cofactor and cofactor analogs: implications for the reaction mechanism. *J. Mol. Biol.* **289**, 277–291 (1999). 10.1006/jmbi.1999.2788 10366505

