# Supplementary material for: Elucidating the multiple roles of hydration for accurate protein-ligand binding prediction via deep learning
Source: Commun Chem. 2020 Feb 11;3:19. doi: 10.1038/s42004-020-0261-x (PMC9814895; doi:10.1038/s42004-020-0261-x)
Supplement: Supplementary file 1 — Supplementary Information [file 42004_2020_261_MOESM1_ESM.pdf]

Supplementary Methods

MD Simulations

MD simulations were performed using the modified GPU-accelerated OpenMM-WATsite package with the AMBER14SB force field [1] and SPC/E water model [2, 3]. The SHAKE algorithm [4] was applied to constrain bonds including hydrogen atoms to their equilibrium lengths and maintain rigid water geometries. Long-range electrostatic interactions were treated with the Particle Mesh Ewald method [5] with a cutoff of 10 Å for the direct interactions. The Lennard-Jones interactions were truncated at a distance of 10 Å, and a long-range isotropic correction was applied to the pressure representing Lennard-Jones interactions beyond the cutoff. A Langevin integrator with a time step of 2 fs was used together with a stochastic thermostat collision frequency of 1 ps^{-1}. The pressure control was implemented via isotropic box edge adjustment by MC moves every 25 time steps simulating the effect of constant pressure. The system was first energy minimized and then heated to 298 K over 50 ps of MD simulations, followed by 1 ns of equilibration MD simulations at 298 K and 1 bar with periodic boundary conditions in all three dimensions. During the minimization and equilibration process, all protein heavy atoms were harmonically restrained with a spring constant of 4.8 kcal mol^{-1} $Å^{-2}$.

Convergence of Grid-Based Energy Analysis

We performed convergence studies of the grid-based energy analysis. 100 ns MD simulations were performed for the production run for the full protein system, and snapshots were saved every picosecond in NetCDF format, generating 100,000 frames. To test the convergence of water-energy calculation, we performed grid-based hydration analysis for the first 1, 2, 3, 4, 5, 10, 20, and 50 ns of the 100 ns MD simulation. Energy grids predicted from shorter simulations were compared to that from 100 ns by calculating the Pearson correlation coefficient r^2 between two sets of energy calculations. r^2 values for ΔG grids (when compared to the full 100 ns simulation results) increase from 0.84 over 0.91 to 0.94 when extending the analysis from the first 1 ns over 3 ns to 5 ns of the MD simulation (Supplementary Table 2). Based on this study, WATsite simulations were performed for 5 ns for each protein system.

Efficiency Measures of Accelerated WATsite3.0 Implementation

To prove that our new accelerated WATsite3.0 implementation is efficient enough for big-data applications, we compared the simulation performance of WATsite3.0 with the previous WATsite2.0 version using GROMACS, here on the DHFR and HIV-1 protease system. When considering just the MD simulation portion itself, a three-fold speed increase is achieved using a single GPU (NVIDIA GTX 1080 Ti) with OpenMM compared to using 16 CPU cores with GROMACS (Supplementary Figure 1A). In addition, the total computation time includes post-analysis for hydration site prediction. The previous WATsite2.0 implementation based on GROMACS performs free energy calculation for each individual hydration site afterwards in the form of an energy rerun. In contrast, the new implementation of WATsite3.0 based on OpenMM generates water energies during the MD simulation. Whereas this implementation slightly reduces the performance of the OpenMM-Watsite simulation compared to standard OpenMM (cf. Supplementary Figure 1A, Equ vs Prod), it accelerates the energy analysis significantly. A speed-up of 15-18 can therefore be obtained for the whole hydration site profiling procedure using WATsite3.0 compared to WATsite2.0 (Supplementary Figure 1B).

Supplementary Tables

PF00246, PF05173, PF00933, PF00494, PF00064, PF00561, PF02036, PF00194, PF11713, PF10502, PF00294, PF00452, PF02142, PF13343, PF01887, PF00393, PF01915, PF02098, PF00885, PF01746, PF00185, PF07748, PF13532, PF00639, PF02542, PF02833, PF02782, PF10431, PF09173, PF06433, PF03099, PF00168, PF11598, PF00275, PF04008, PF03171, PF13377, PF00004, PF01401, PF00343, PF02087, PF14598, PF00342, PF00617, PF00628, PF13419, PF00551, PF00650, PF00077, PF16317, PF00496, PF03331, PF00809, PF07654, PF00583, PF00121, PF00209, PF01513, PF00254, PF03466, PF01808, PF13088, PF02768, PF01220, PF07690, PF02146, PF02984, PF01487, PF13649, PF00718, PF03284, PF00483, PF04587, PF12974, PF01116, PF01431, PF02931, PF00607, PF00354, PF02866, PF00054, PF00144, PF10609, PF13463, PF03767, PF00557, PF00156, PF07555, PF17387, PF08450, PF09041, PF00450, PF03414, PF01156, PF01344, PF02263, PF05014, PF08832, PF16591, PF02608, PF02743, PF00017, PF00334, PF00155, PF00413, PF02780, PF03143, PF08534, PF09515, PF00152, PF03129, PF00682, PF00516, PF04057, PF16355, PF01094, PF02156, PF00731, PF02982, PF01380, PF07971, PF00543, PF00113, PF05049, PF09127, PF01361, PF17209, PF01596, PF00653, PF02097, PF02991, PF00186, PF00676, PF00215, PF16518, PF14681, PF02502, PF08778, PF08915, PF07109, PF14670, PF00962, PF00850, PF00439, PF01419, PF01633, PF00604, PF03352, PF00398, PF01234, PF01023, PF02171, PF00701, PF00959, PF07081, PF00027, PF00576, PF00061, PF00501, PF13688, PF03308, PF02868, PF05336, PF00331, PF00071, PF00188, PF01557, PF03740, PF01187, PF10613, PF01702, PF01979, PF07472, PF00102, PF01327, PF03401, PF00703, PF08761, PF02518, PF01575, PF07714, PF02449, PF00781, PF01661, PF02567, PF00857, PF00656, PF00291, PF01255, PF05191, PF00998, PF00075, PF04389, PF00068, PF00092, PF01547, PF07311, PF00104, PF07737, PF07828, PF03941, PF01555, PF02254, PF02274, PF00069, PF01163, PF00293, PF00351, PF04198, PF01288, PF17432, PF02569, PF00025, PF12296, PF00001, PF02223, PF14509, PF00160, PF02492, PF02785, PF13499, PF01653, PF01244, PF00106, PF00043, PF06441, PF06479, PF01636, PF00693, PF00590, PF08502, PF05351, PF00692, PF11485, PF00337, PF14714, PF00775, PF00183, PF00728, PF02136, PF00407, PF07686, PF00440, PF00491, PF01728, PF00348, PF00685, PF00193, PF00591, PF08211, PF00169, PF00051, PF00218, PF01120, PF14497, PF01048, PF01204, PF08544, PF13202, PF00232, PF00870, PF13193, PF16849, PF02775, PF03193, PF00702, PF00497, PF01195, PF00162, PF00233, PF00150, PF00704, PF05726, PF01053, PF00026, PF00977, PF07662, PF09359, PF13354, PF13407, PF01042, PF09273, PF01395, PF03246, PF01202, PF01532, PF00588, PF01382, PF00139, PF02201, PF00204, PF12697, PF02041, PF09458, PF04069, PF08411, PF00821, PF00171, PF08447, PF00180, PF02574, PF08123, PF12680, PF00326, PF13416, PF00425, PF02801, PF03480, PF00079, PF00691, PF00753, PF07977, PF17211, PF00456, PF00679, PF02816, PF01663, PF17189, PF11941, PF00135, PF13458, PF05053, PF00062, PF00274, PF01421, PF08423, PF00903, PF13640, PF02827, PF04253, PF00793, PF00532, PF08541, PF02237, PF03306, PF17645, PF11838, PF03024, PF03328, PF02774, PF00112, PF00202, PF13561, PF00644, PF00132, PF13714, PF16186, PF04095, PF13545, PF00303, PF08543, PF04209, PF00408, PF07615, PF00665, PF09396, PF00012, PF12919, PF00161, PF00248, PF13378, PF01473, PF00545, PF02421, PF00074, PF00089, PF00579, PF00426, PF09160

Supplementary Table 1: List of PFAM families in test set.

	r^2			
Checkpoint (ns)	Occupancy	ΔG	ΔH	-TΔS
1	0.98	0.84	0.85	0.85
2	0.99	0.89	0.90	0.89
3	0.99	0.91	0.91	0.91
4	0.99	0.93	0.94	0.93
5	0.99	0.94	0.94	0.94
7	1.00	0.95	0.96	0.95
10	1.00	0.96	0.97	0.96
20	1.00	0.97	0.98	0.98
50	1.00	0.99	0.99	0.99

Supplementary Table 2: Pearson correlation coefficient r^2 for occupancy and energy grids of HIV-1 protease at different simulation lengths compared to simulation with a total of 100 ns.

	OC				
Truncation (Å)	Occupancy	ΔG	ΔH	-TΔS	Speed (ns/day)
12	0.97	0.91	0.91	0.93	255
15	0.97	0.91	0.91	0.93	214
17	0.97	0.92	0.91	0.94	189
20	0.97	0.93	0.93	0.95	173
25	0.97	0.93	0.93	0.95	173
30	1.00	1.00	1.00	1.00	173

Supplementary Table 3: Effects of truncation on the accuracy of grid energies and the speed of simulation.

Supplementary Figures

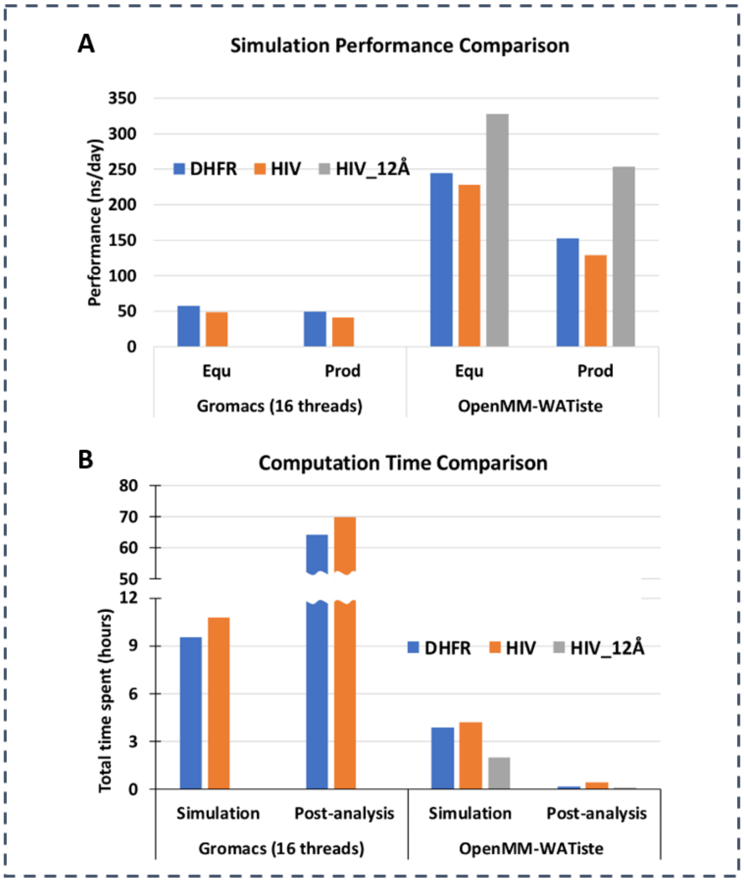

Supplementary Figure 1: Simulation performance of WATsite with and without GPU-acceleration. (A) Performance measured in ns/day for simulating DHFR (blue), HIV-1 protease without (orange) and with 12 Å threshold radius (gray) using Gromacs on 16 threads and using OpenMM-WATsite without (Equ) and with water energy calculation and output (Prod). (B) Total time spent on 20 ns MD simulation separated by simulation and post-analysis.

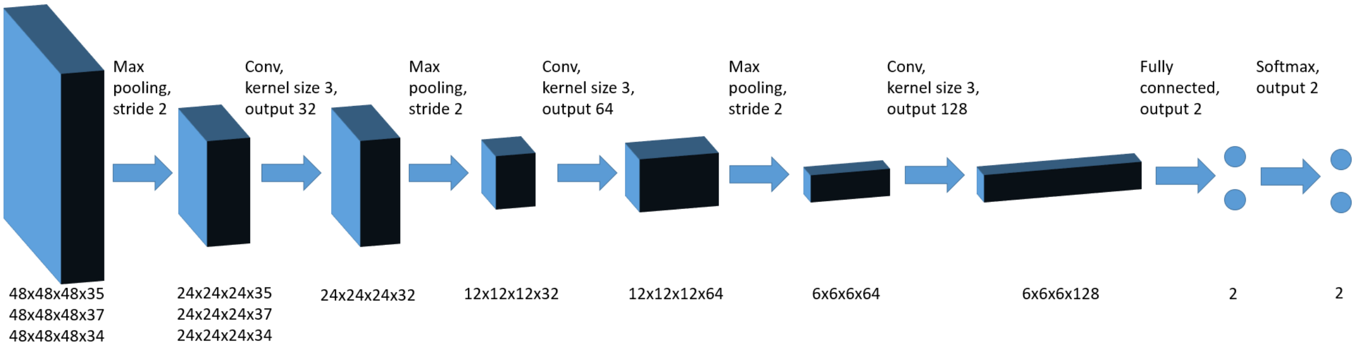

Supplementary Figure 2: Architecture of CNNs used in this study.

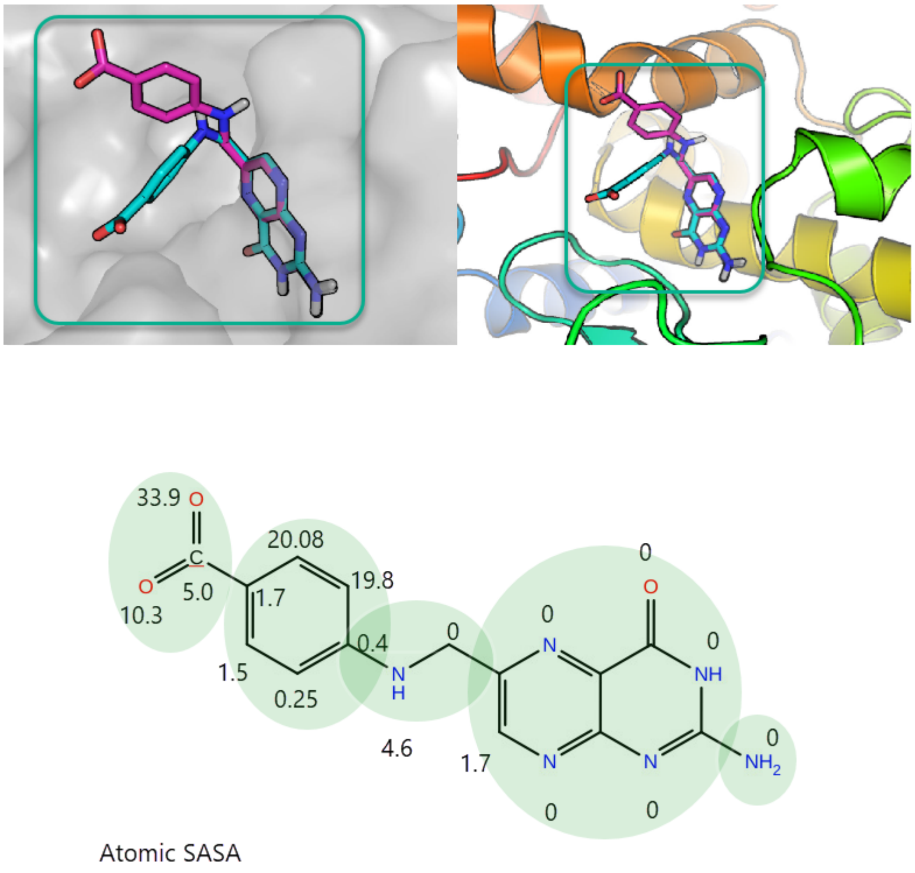

Supplementary Figure 3: Example for modified RMSD calculation: Atomic SASA for a givenn ligand together with the fragmentation pattern used to calculate the modified RMSD.

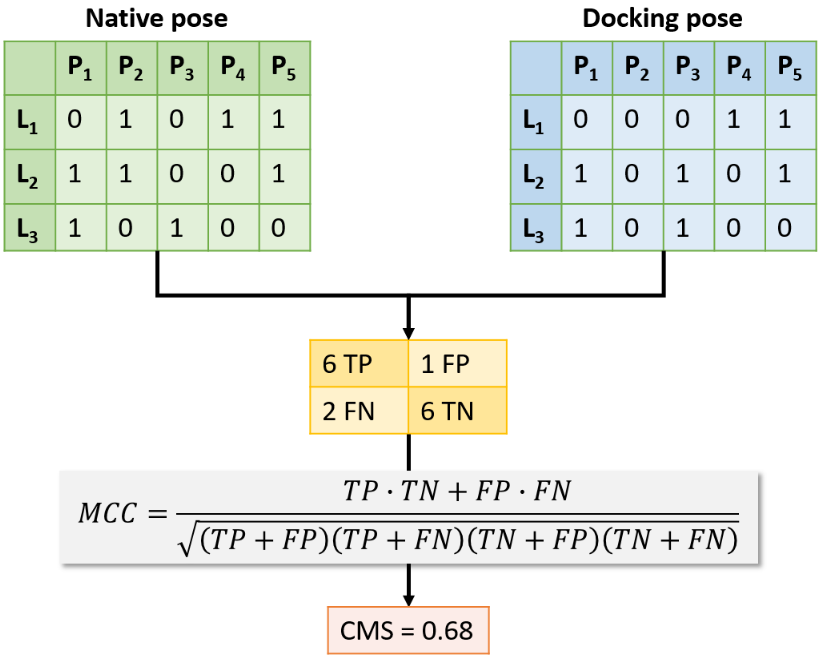

Supplementary Figure 4: CMS is computed using Matthews Correlation Coefficient (MCC) of confusion matrix comparing protein-ligand contacts of docked with native pose (adapted from [6]).

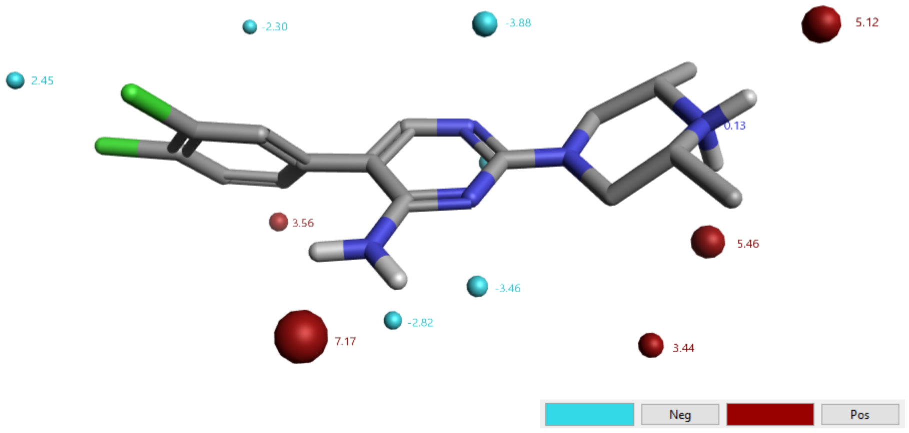

Supplementary Figure 5: Example of extrema values for electrostatic interactions with negative and positive probe using the XED force field.

Supplementary Notes

Supplementary Note 1. Short Review on the Treatment of Hydration in Past and Current Docking Concepts

In the past, the inclusion of hydration effects in docking software were focused on the modeling of desolvation effects [7]. This effect was typically modelled by empirical terms characterizing hydrophobic contacts between the protein and the ligand, e.g. using counts of close hydrophobic protein-ligand atom pairs or using a term that is proportional to the solvent-accessible surface. In X-Score [8], for example, three different strategies were tested to model the hydrophobic effect which is based on protein and ligand desolvation. In the hydrophobic contact strategy (X-ScoreHC), the number of protein-ligand atom pairs containing hydrophobic atom types are computed, in the hydrophobic matching strategy (X-ScoreHM) the logP values of the atoms are considered, and in the hydrophobic surface strategy (X-ScoreHS), the ligand hydrophobic surface in contact with the solvent accessible surface of the protein is computed. A more sophisticated term representing protein shape by a curvature-dependent surface-area term in Cyscore [9] lead to significant improvement in affinity prediction.

Whereas those approaches focus on the favorable contribution of desolvation in the context of the hydrophobic effect, unfavorable desolvation effect also can significantly contribute to protein-ligand thermodynamics, e.g. burying polar groups upon protein-ligand association. Specific penalization terms have been developed and incorporated in various scoring functions, such as in ICM [10], XP GlideScore [11], LigScore [12], and DockTScore [13].

Another class of (de)solvation models adapted to docking are based on physics-based implicit solvation models, in particular MM-PBSA and MM-GBSA approaches. Sun et al. [14] successfully applied MM-GBSA for free energy estimation using docking to a large number of protein–ligand complexes. Mysinger et al. [15] implemented a simplified GBSA scheme into the DOCK program. A damped version of MM-PBSA was successfully integrated in the HADDOCK scoring function for binding pose and affinity prediction of protein–peptide complexes [16].

All these previously discussed approaches implicitly model desolvation effects. They, however, ignore the individual thermodynamic properties of binding-site water molecules which differ based on the protein environment. To explicitly and more precisely model those water molecules, a spectrum of various computational approaches have been developed to identify the likely position of water molecules in binding sites and to evaluate their energetic stability [17].

Different strategies have been devised to incorporate explicit water molecules into docking programs. One class of approaches focused on the inclusion of explicit water molecules critical for mediating protein-ligand interactions. In the simplest approaches binding-site water molecules, either predicted or from X-ray structures, are manually selected and are kept throughout docking as part of the protein template [18]. Based on the selection process and dependent on the studied protein system increase but also decrease in docking quality were identified. For a flexible treatment of water-mediated interactions different adaptation of widely-used docking programs have been devised. For example in GOLD [19] water molecules are switched on and off and are allowed to rotate throughout the docking process. In AutoDock [20] water molecules are attached to the docked ligand and their turning on or off is evaluated during the scoring process for each given docking pose. The energetic evaluation of the individual water molecules is, however, still based on empirical scoring terms.

For a more accurate estimation of desolvation energies of individual water molecules, MC or MD-based methods, such as WaterMAP [21, 22] or WATsite [23, 24] were developed. For a direct inclusion of explicit desolvation into standard scoring procedures, grid-based adaptations of the inhomogeneous solvation theory (IST)[25, 26], such as GIST[27], were utilized. Even with such accurate desolvation estimation, the docking performance did not significantly nor consistently improve for most protein targets [28]. Reasons for these results include the lack of consistent scoring function design and optimization including the explicit desolvation term (desolvation term has been typically used as subsequent add-on to existing scoring functions) or inaccuracies in the other interactions terms which are typically simplified empirical representation for the underlying interactions.

Supplementary Note 2. Analysis by Protein Family

To investigate whether the observed performance gain of DeepWATsite is independent of the protein family or if there are specific families where the model did not outperform standard scoring approaches, the model performance was separately analyzed by protein family (using PFAM classification). The supplementary files Supplementary_Data_3.csv

and Supplementary_Data_4.csv displays the results for all families and those with at least 20 complex structures in the data set. The file contains PFAM key, PFAM name, PFAM description, total number of complexes in data set, number of Smina failures (top-1), machine learning failures (top-1) using DeepWATsite without corrections due to solvent accessibility and low occupancy, machine learning failures (top-1) using DeepWATsite with correction due to solvent accessibility and low occupancy. The analysis revealed that DeepWATsite, even without correction for solvent accessibility and low occupancy, displays enhanced performance for almost all families. Including the corresponding correction in the loss function resulted in a model that improves performance for all families in the data set. Despite this enhancement, some families still show non-optimal performance, e.g. for families with fragment-sized ligands and those with large, flexible hydrophobic tails.

Supplementary Note 3. Comparison with Other Shallow and Deep Learning Approaches

To compare the results from DeepWATsite with those of other machine learning methods, we applied several shallow and deep learning techniques onto the exact same training (only large training set) and test set. From a deep learning perspective, completely different featurization techniques were tested ranging from grid featurization, PointNet representation to graph-based algorithms. All methods were tested for re-scoring performance, i.e. the poses retrieved from Smina were used as input, as described in section S3.1. It should be noted that in the current study, the test set comprised a diverse subset of 369 families (based on PFAM classification). All systems were exposed to a normal docking procedure using Smina unlike other synthetic benchmark sets where the docking poses are artifically balanced using RMSD binning [29]. Our procedure guarantees that each system has the natural unbalance in the data that is observed in practical docking experiments where near native poses are far less observed compared to the decoys. Furthermore, we do not omit so-called borderline poses with RMSD in the range between 2 Å and 4 Å a standard procedure in similar studies using machine learning methods, e.g. [30]. Our strategy leads to more difficult model training and validation, as those border-line poses are difficult to classify using machine learning methods. Ignoring those poses boost the documented performance, however, this does not adequately reflect model performance in real-life scenarios where the native pose is unknown and omission of borderline poses is therefore impossible.

**Method**	**Prediction accuracy (top-1)**	Reference
Smina/Vina	63%	[31]
$\Delta_{vina}RF_{20}$	61%	[32]
PointNet	63%	[33]
PointNet++	65%	[34]
Graph Convolution with Ranking	54%	[35]
Graph Attention (GCN-DTI)	81%	[36]
CNN	70%	This study; [30]
DeepWATsite	89%	This study

Supplementary Table 4: Docking Power Benchmark using various Deep-learning methods on test set.

Supplementary Table 4 displays the test set results for the initial docking results using classical scoring function Smina/Vina [31], a random forrest method $\Delta_{vina}RF_{20}$ [32], initial PointNet [33] and PointNet++ featuring hierarchical point representation [34], two different graph convolution methods[35, 36], CNN based on protein and ligand density [30], and the final DeepWATsite model. Whereas $\Delta_{vina}RF_{20}$, the PointNet models and one graph convolution network performs similar to the classical scoring functions, only CNN and in particular the graph convolution method GCN-DTI boosts the pose-ranking performance. None of the methods, however, approaches the accuracy observed for DeepWATsite.

References

[1] James A. Maier, Carmenza Martinez, Koushik Kasavajhala, Lauren Wickstrom, Kevin E. Hauser, and Carlos Simmerling. ff14sb: Improving the accuracy of protein side chain and backbone parameters from ff99sb. *J. Chem. Theory Comput.*, 11(8):3696–3713, 2015.

[2] H. J. C. Berendsen, J. R. Grigera, and T. P. Straatsma. The missing term in effective pair potentials. *J. Phys. Chem.*, 91(24):6269–6271, 1987.

[3] Swaroop Chatterjee, Pablo G. Debenedetti, Frank H. Stillinger, and Ruth M. Lynden-Bell. A computational investigation of thermodynamics, structure, dynamics and solvation behavior in modified water models. *J. Phys. Chem.*, 128(12):124511, 2008.

[4] Jean-Paul Ryckaert, Giovanni Ciccotti, and Herman J.C Berendsen. Numerical integration of the cartesian equations of motion of a system with constraints: molecular dynamics of n-alkanes. *J. Comput. Phys.*, 23(3):327–341, 1977.

[5] T. Darden, D. York, and L. Pedersen. Particle mesh Ewald: An Nlog(N) method for Ewald sums in large systems. *J. Chem. Phys.*, 98:10089–10092, 1993.

[6] Yun Ding, Ye Fang, Juana Moreno, J. Ramanujam, Mark Jarrell, and Michal Brylinski. Assessing the similarity of ligand binding conformations with the contact mode score. *Comput. Biol. Chem.*, 64:403 – 413, 2016.

[7] Markus A. Lill. Efficient incorporation of protein flexibility and dynamics into molecular docking simulations. *Biochemistry*, 50(28):6157–6169, 2011.

[8] Renxiao Wang, Luhua Lai, and Shaomeng Wang. Further development and validation of empirical scoring functions for structure-based binding affinity prediction. *J. Comput.-Aided Mol. Des.*, 16(1):11–26, 2002.

[9] Yang Cao and Lei Li. Improved protein–ligand binding affinity prediction by using a curvature-dependent surface-area model. *Bioinformatics*, 30(12):1674–1680, 2014.

[10] Maxim Totrov and Ruben Abagyan. Derivation of sensitive discrimination potential for virtual ligand screening. In *Proceedings of the third annual international conference on Computational molecular biology - RECOMB 99*. ACM Press, 1999.

[11] Richard A. Friesner, Robert B. Murphy, Matthew P. Repasky, Leah L. Frye, Jeremy R. Greenwood, Thomas A. Halgren, Paul C. Sanschagrin, and Daniel T. Mainz. Extra precision glide: docking and scoring incorporating a model of hydrophobic enclosure for protein-ligand complexes. *J. Med. Chem.*, 49(21):6177–6196, 2006.

[12] André Krammer, Paul D. Kirchhoff, X. Jiang, C.M. Venkatachalam, and Marvin Waldman. LigScore: a novel scoring function for predicting binding affinities. *J. Mol. Graphics Modell.*, 23(5):395–407, 2005.

[13] Camila Silva de Magalhães, Diogo Marinho Almeida, Helio José Correa Barbosa, and Laurent Emmanuel Dardenne. A dynamic niching genetic algorithm strategy for docking highly flexible ligands. *Inf. Sci.*, 289:206–224, 2014.

[14] Huiyong Sun, Youyong Li, Mingyun Shen, Sheng Tian, Lei Xu, Peichen Pan, Yan Guan, and Tingjun Hou. Assessing the performance of MM/PBSA and MM/GBSA methods. 5. improved docking performance using high solute dielectric constant MM/GBSA and MM/PBSA rescoring. *Phys. Chem. Chem. Phys.*, 16(40):22035–22045, 2014.

[15] Michael M. Mysinger and Brian K. Shoichet. Rapid context-dependent ligand desolvation in molecular docking. *J. Chem. Inf. Model.*, 50(9):1561–1573, 2010.

[16] Dimitrios Spiliotopoulos, Panagiotis L. Kastritis, Adrien S. J. Melquiond, Alexandre M. J. J. Bonvin, Giovanna Musco, Walter Rocchia, and Andrea Spitaleri. dMM-PBSA: A new HADDOCK scoring function for protein-peptide docking. *Front. Mol. Biosci.*, 3:46, 2016.

[17] Eva Nittinger, Florian Flachsenberg, Stefan Bietz, Gudrun Lange, Robert Klein, and Matthias Rarey. Placement of water molecules in protein structures: From large-scale evaluations to single-case examples. *J. Chem. Inf. Model.*, 58(8):1625–1637, 2018.

[18] Ashutosh Kumar and Kam Y. J. Zhang. Investigation on the effect of key water molecules on docking performance in csardock exercise. *J. Chem. Inf. Model.*, 53(8):1880–1892, 2013.

[19] M L Verdonk, G Chessari, J C Cole, M J Hartshorn, C W Murray, J W Nissink, R D Taylor, and R Taylor. Modeling water molecules in protein-ligand docking using GOLD. *J. Med. Chem.*, 48(20):6504–6515, 2005.

[20] Stefano Forli and Arthur J. Olson. A force field with discrete displaceable waters and desolvation entropy for hydrated ligand docking. *J. Med. Chem.*, 55(2):623–638, 2012.

[21] Robert Abel, Tom Young, Ramy Farid, Bruce J. Berne, and Richard A. Friesner. Role of the active-site solvent in the thermodynamics of factor xa ligand binding. *J. Am. Chem. Soc.*, 130(9):2817–2831, 2008.

[22] Tom Young, Robert Abel, Byungchan Kim, Bruce J. Berne, and Richard A. Friesner. Motifs for molecular recognition exploiting hydrophobic enclosure in protein–ligand binding. *P. Natl. Acad. Sci. USA*, 104(3):808–813, 2007.

[23] Bingjie Hu and Markus A. Lill. Watsite: Hydration site prediction program with pymol interface. *J. Comput. Chem.*, 35(16):1255–1260, 2014.

[24] Ying Yang, Bingjie Hu, and Markus A. Lill. Watsite2.0 with pymol plugin: Hydration site prediction and visualization. In *Methods Mol. Biol. (N.Y., NY, U.S.)*, pages 123–134. Springer, 2017.

[25] Themis Lazaridis. Inhomogeneous fluid approach to solvation thermodynamics. 1. theory. *J. Phys. Chem. B*, 102(18):3531–3541, 1998.

[26] Themis Lazaridis. Inhomogeneous fluid approach to solvation thermodynamics. 2. applications to simple fluids. *J. Phys. Chem. B*, 102(18):3542–3550, 1998.

[27] Crystal N Nguyen, Tom Kurtzman Young, and Michael K Gilson. Grid inhomogeneous solvation theory: hydration structure and thermodynamics of the miniature receptor cucurbit[7]uril. *J. Chem. Phys.*, 137(4):044101, 2012.

[28] Trent E. Balius, Marcus Fischer, Reed M. Stein, Thomas B. Adler, Crystal N. Nguyen, Anthony Cruz, Michael K. Gilson, Tom Kurtzman, and Brian K. Shoichet. Testing inhomogeneous solvation theory in structure-based ligand discovery. *Proc. Natl. Acad. Sci. U.S.A.*, 114(33):E6839–E6846, 2017.

[29] Yan Li, Li Han, Zhihai Liu, and Renxiao Wang. Comparative assessment of scoring functions on an updated benchmark: 2. evaluation methods and general results. *J. Chem. Inf. Model.*, 54(6):1717–1736, 2014.

[30] Matthew Ragoza, Joshua Hochuli, Elisa Idrobo, Jocelyn Sunseri, and David Ryan Koes. Protein–Ligand Scoring with Convolutional Neural Networks. *J. Chem. Inf. Model.*, 57(4):942–957, 2017.

[31] David Ryan Koes, Matthew P Baumgartner, and Carlos J Camacho. Lessons learned in empirical scoring with smina from the csar 2011 benchmarking exercise. *J. Chem. Inf. Model.*, 53(8):1893–1904, 2013.

[32] Cheng Wang and Yingkai Zhang. Improving scoring-docking-screening powers of protein–ligand scoring functions using random forest. *J. Comput. Chem.*, 38(3):169–177, 2017.

[33] Charles R Qi, Hao Su, Kaichun Mo, and Leonidas J Guibas. Pointnet: Deep learning on point sets for 3d classification and segmentation. In *Proceedings of the IEEE Conference on Computer Vision and Pattern Recognition*, pages 652–660, 2017.

[34] Charles Ruizhongtai Qi, Li Yi, Hao Su, and Leonidas J Guibas. Pointnet++: Deep hierarchical feature learning on point sets in a metric space. In *Advances in neural information processing systems*, pages 5099–5108, 2017.

[35] Joseph A Morrone, Jeffrey K Weber, Tien Huynh, Heng Luo, and Wendy D Cornell. Combining docking pose rank and structure with deep learning improves protein-ligand binding mode prediction. *arXiv preprint arXiv:1910.02845*, 2019.

[36] Jaechang Lim, Seongok Ryu, Kyubyong Park, Yo Joong Choe, Jiyeon Ham, and Woo Youn Kim. Predicting drug–target interaction using a novel graph neural network with 3d structure-embedded graph representation. *J. Chem. Inf. Model.*, 59(9):3981–3988, 2019.
