# Supplementary material for: Elucidating the multiple roles of hydration for accurate protein-ligand binding prediction via deep learning
Source: Commun Chem. 2020 Feb 11;3:19. doi: 10.1038/s42004-020-0261-x (PMC9814895; doi:10.1038/s42004-020-0261-x)
Supplement: Supplementary file 2 — Description of Additional Supplementary Files [file 42004_2020_261_MOESM2_ESM.pdf]

Description of Additional Supplementary Files

File Name: Supplementary Data 1
Description: Systems containing ligands with low occupancy in corresponding X-ray complex structure.

File Name: Supplementary Data 2
Description: Systems used in training sets, test set and cross-validation sets.

File Name: Supplementary Data 3
Description: Failure rate of Smina, DeepWATsite and DeepWATsite with corrections for solvent-accessibility and low occupancy for all protein families in dataset.

File Name: Supplementary Data 4
Description: Failure rate of Smina, DeepWATsite and DeepWATsite with corrections for solvent-accessibility and low occupancy for protein families in dataset with at least 20 complexes per family.
